# Integrated profiling of metaplastic breast cancer identifies putative master regulators of intratumoral heterogeneity

**DOI:** 10.1038/s41523-025-00807-x

**Published:** 2025-08-11

**Authors:** Yufan Feng, Albert Xiong, Onkar Mulay, Anna Sokolova, Malcolm Lim, Benjamin Van Haeringen, Natasha McGuire, Xavier de Luca, Peter T. Simpson, Quan Nguyen, Sunil R. Lakhani, Amy E. McCart Reed

**Affiliations:** 1 UQ Centre for Clinical Research, Faculty of Health, Medicine and Behavioural Sciences, The University of Queensland, Brisbane, 4029 Australia; 2 QIMR Berghofer Medical Research Institute, Brisbane, 4006 Australia; 3 Institute for Molecular Bioscience, The University of Queensland, Brisbane, 4029 Australia; 4 Sullivan Nicolaides Pathology, Bowen Hills, Brisbane, 4006 Australia; 5 Pathology Queensland, The Royal Brisbane and Women’s Hospital, Brisbane, 4029 Australia; 6 School of Biomedical Sciences, Faculty of Health, Medicine and Behavioural Sciences, The University of Queensland, Brisbane, Australia

**Keywords:** Breast cancer, Cancer genomics

## Abstract

Metaplastic breast cancer (MpBC) is defined by the presence of various morphological elements, typically biphasic, with epithelial (e.g. no-special type (NST), squamous) and mesenchymal (e.g. spindle, chondroid, osteoid) components. The established clonality of the different components favours an evolution model encompassing either a multipotent progenitor, or a linear metaplastic conversion. We used methylation profiling and showed that different morphologies have specific methylation profiles. Furthermore, our spatial transcriptomic approach, using 10× Genomics Visium and trajectory analysis, evidenced that spindle cells form a transition between the originating carcinoma of no-special type (NST) and pleomorphic regions, with osteoid differentiation likely to be an end-stage fate of the chondroid growth pattern, supporting the conversion model of lineage differentiation. We have also identified a series of master transcription factors likely to regulate these processes, and are significantly associated with metaplastic-like clinical features. This data further supports the conversion model of metaplasia and warrants functional analysis.

## Introduction

Metaplastic breast cancer (MpBC) is a distinct subtype of breast cancer (BC) characterized by morphological diversity and significantly increased mortality compared to other BC subtypes^1^. MpBC are typically Estrogen Receptor (ER) and Progesterone Receptor (PR) negative, and lack HER2 overexpression, making them ‘triple negative’ (TNBC). By gene expression profiling, MpBCs are classed as ‘claudin-low’, a type of basal-like BC consistent with strong epithelial to mesenchymal transition (EMT) pathway activation^2^. MpBC have regions of carcinoma of no-special type (NST) and exhibit differentiation into heterologous elements such as squamous, spindle, chondroid, and osteoid^1^. MpBC respond poorly to chemotherapy, and due to the absence of effective targeted therapy, management can be challenging^3^.

The distinct morphologies in MpBC have different prognostic implications with high-grade spindle and mixed metaplastic carcinoma having the worst prognosis, and squamous carcinoma phenotype has a more favourable prognosis^4,5^. The increasing degree of morphologic heterogeneity is also associated with a poorer outcome^5^.

Morphologically distinct subpopulations within a single tumour display similar genetic and immunohistochemical profiles^6^. Spindle, squamous, and chondroid morphologies have largely similar copy number alteration patterns^7^. Whole-exome sequencing of eight MpBC cases revealed a common genomic ancestry between metaplastic elements^8^ and transcriptomics revealed that the within a tumour, the NST or undifferentiated component, expressed signatures related to the heterologous elements^9^. Together, these studies indicate that the morphologic components within a case are clonally-related, but also that the diversity is not driven by the acquisition of alterations in the evolving cancer genome. Whether these morphologies dynamically transition during tumour progression is uncertain. Indeed, there are two theories that can explain the clonal origins of MpBC: firstly, that there is a multipotent progenitor cell from which originate the different phenotypes directly; and secondly, that there is linear metaplastic conversion along a spectrum of phenotypes.

Epigenetic regulation in normal tissue development is critical for proper lineage commitment, cell fate determination, and organogenesis. We therefore consider that genome methylation changes might play a role in driving tumour plasticity in MpBC. Here we present the application of methylation profiling tied with spatial transcriptomics to investigate the mechanisms driving metaplasia and heterogeneity in MpBC.

## Results

### Profiling of Methylation Patterns in Distinct MpBC Morphologies

**Fig. 1:**
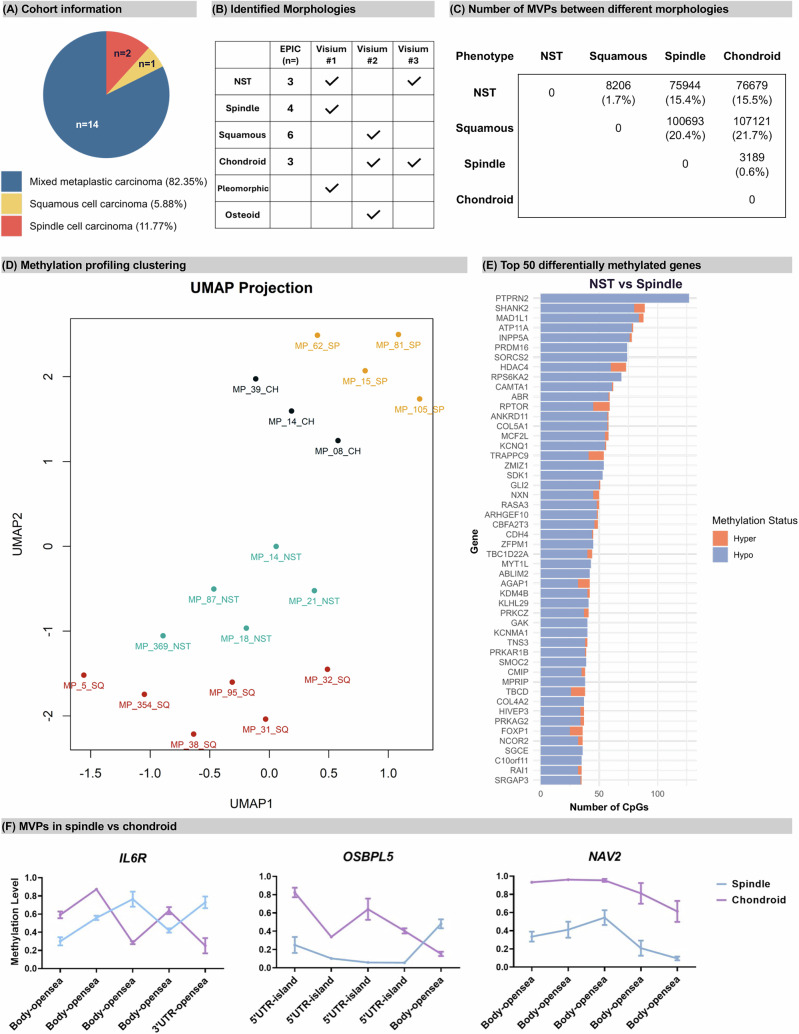
Overview of Methylation Profiling in Metaplastic Breast Cancer. **A** Cohort Information. **B** Breakdown of morphologies analysed with Infinium MethylationEPIC profiling (EPIC) and 10× Genomics Visium spatial transcriptomics. **C** Number of significant Methylation-Variable Positions (MVPs) identified in comparisons between distinct morphologies. **D** UMAP clustering projection plot for all detected CpG sites across the cohort (*n* = 18). **E** Bar plot displaying the top 50 genes enriched with significantly differentially methylated CpGs for NST vs Spindle. The horizontal axis represents the number of significant MVPs. Methylation status: ‘Hyper’ and ‘Hypo’ indicate hypermethylation or hypomethylation in the NST cohort compared to spindle. **F** MVPs for *IL6R*, *OSBPL5*, and *NAV2* in Spindle vs. Chondroid plotted as the genomic location of the MVPs, against the methylation β level.

To profile subregions of MpBC, we used needle enrichment dissection. DNA methylation was profiled using Infinium MethylationEPIC v1.0 arrays from 32 samples (31 cases), 18 samples (including both NST and chondroid from MP_14) passed quality control (QC) and among these, 14 (representing 82.35% of cases) were classified as mixed MpBC, one case (5.88%) as ‘pure’ squamous, and two cases (11.77%) as ‘pure’ spindle (Fig. 1A). After QC filtering, 494,046 probes (p < 0.01) remained for comparison between morphologies. The morphologies selected included carcinoma of no special type (NST), squamous, spindle, and chondroid (Fig. 1B) and each morphology was compared against the other groups to identify significant methylation variable positions (MVPs) using linear modelling (|Δβ | >0.2; p-values < 0.01). The number and proportion of MVPs among all detected CpGs are shown in Fig. 1C. We observed significantly fewer MVPs when comparing NST to squamous (8206 MVPs; 1.7%), and spindle to chondroid (3,189 MVPs; 0.6%), indicating relatively similar methylation patterns (Fig. 1C). Conversely, more MVPs were noted in comparisons between epithelial morphologies (NST or squamous) versus mesenchymal morphologies (spindle or chondroid). UMAP (Uniform Manifold Approximation and Projection) visualization (Fig. 1D) and unsupervised hierarchical clustering (Supplementary Fig. 1) demonstrated stratification of samples. In addition, Supplementary Fig. 1 shows that intra-patient heterogeneity in MpBCs remains considerable, even when samples with the same morphological phenotype cluster together.

The top 50 MPVs, and their status as hyper- or hypo-methylated (|Δβ | >0.2, *p* < 0.01), are shown in Supplementary Fig. 2, with the CpG localization shown in Supplementary Fig. 3. In NST versus squamous, *KIF26B* is the most differentially methylated gene with 36 MVPs followed by *SEPT9* (MVPs=21) (Supplementary Fig. 2A). Comparing NST to spindle and NST to chondroid, most MVPs were hyper-methylated in the spindle or chondroid components (i.e. hypomethylated in NST; Fig. 1E, Supplementary Fig. 2). The most differentially methylated genes across comparisons with NST or squamous included *PTPRN2*, *SHANK2*, *MAD1L1* and *HDAC4* (Fig. 1E, Supplementary Fig. 2C–E), with upwards of >79 MVPs dependent on comparison. Between spindle and chondroid, *SORCS2* and *ZFPM1* had the highest number of MVPs (Supplementary Fig. 2F). Most genes were hypermethylated in spindle compared to chondroid, a subset was classed as hypomethylated (Supplementary Fig. 2D) and each had 5 significant MVPs: *IL6R* (3 hypomethylated, 2 hypermethylated); *OSBPL5* (4 hypomethylated, 1 hypermethylated); *NAV2* (all MVPs hypomethylated) (Fig. 1F).

A differentially methylated region (DMR) analysis approach, using the Probe Lasso algorithm (Supplemental Materials), confirmed our CpG analysis, showing similar methylation patterns in NST and squamous, as well as spindle and chondroid (Supplementary Fig. 4A, B; Supplementary Tables 2–7). The average number of MVPs per gene was lower in epithelial vs. epithelial and mesenchymal vs. mesenchymal comparisons than in epithelial vs. mesenchymal comparisons (Supplementary Fig. 4C).

Limited methylation data is available for other specific morphological states examined however, some gene level methylation data was available for squamous cell carcinomas (SCC). Cutaneous SCC shows promoter hypermethylation in genes such as *CDKN2A, CDH1, CDH13, FOXE1, FRZB*, and *DAPK1*^10^. However, our data reveal hypomethylated promoters in these genes across all four morphologies (Supplementary Fig. 5A–F). Specifically, NST and squamous were similar, while chondroid exhibited significant promoter hypomethylation. Methylation of *PAX9, SIM2*, and *THSD4* is a crucial tumour specific event in esophageal SCC (eSCC), with hypermethylation and low expression in eSCC compared to normal esophagus^11^. Supplementary Fig. 5G–I shows hypomethylation in the promoter region in squamous, aligning with normal esophageal tissue. Additionally, for other normal esophagus-specific transcripts like *IL1RN* and *TRIM29*, MpBC squamous also exhibited low methylation levels in the promoter region (Supplementary Fig. 5j, K). In the *TRIM29* promoter, the spindle shows significantly higher methylation levels than NST. This is consistent with the spatial transcriptomics expression results in case one below, where *TRIM29* is downregulated (Log2FC = -1.99, *p* = 0.0002) in spindle compared to NST. Together, the distinctive squamous morphological regions in MpBC may not share the same methylation patterns as squamous cell carcinomas in other organs, and in this analysis are more like normal squamous cells. Chondrosarcomas are subclassified into dedifferentiated chondrosarcoma, clear cell chondrosarcoma, conventional chondrosarcoma and mesenchymal chondrosarcoma subtypes based on morphology and the expression of a series of markers^12^. Supplementary Fig. 6A–H shows some significant promoter hypomethylation in chondroid for markers of dedifferentiated and conventional chondrosarcomas.

### Granular analysis of metaplastic transcriptomes with spatial context

**Fig. 2:**
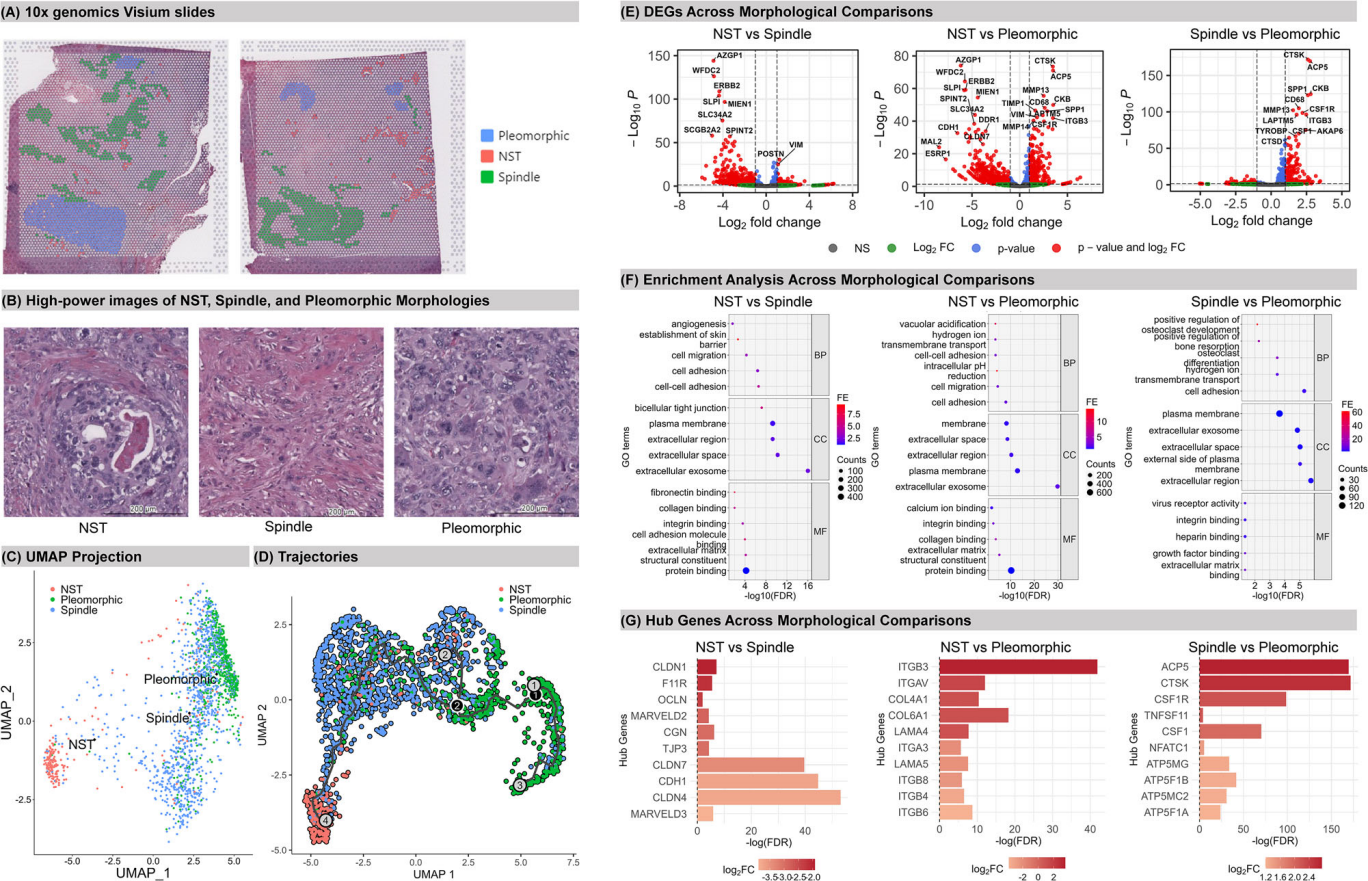
Spatial Transcriptomics Analysis of Case One. **A** Manually annotated distinctive morphologies: invasive carcinoma - no special type (NST), spindle and pleomorphic. The spindle component comprised fascicles of atypical, elongated tumour cells. The pleomorphic component was defined by the presence of highly atypical epithelioid tumour cells demonstrating marked variation in nuclear size and shape, with occasional bizarre tumour giant cells. The pleomorphic component in this case was also associated with osteoclast-like stromal giant cells, which are non-neoplastic cells of histiocytic lineage, demonstrating phenotypic similarity to osteoclasts. The NST component was the malignant carcinomatous component of the tumour that did not demonstrate any special morphologic features. **B** High powered images of phenotypes. **C** Clustering and **D** trajectory analysis plot. Red indicates NST; green represents pleomorphic; blue denotes spindle. The black lines represent the connections between different points or states in the trajectory (each black line represents a branch of a trajectory and each branch has its own outcome). Each light grey circle with a number corresponds to a distinct cell fate (outcome of a branch). Black circles are branching points, where cells can diverge and follow different paths toward various outcomes. The numbers in the black and grey circles are not related to the tissue origin of the cell, but the structure of graph, indicating the branch (paths) and leaves (outcomes of the paths). **E** Volcano plots for differentially expressed genes (DEGs) across three comparisons. The threshold for significant DEGs is adjusted p-value < 0.05 and |Log2FC | >1. Significant DEGs are marked in red; DEGs with adjusted p-value < 0.05 and |Log2FC | <1 are in blue; DEGs with adjusted p-value > 0.05 and |Log2FC | >1 are in green; NS, non-significant. Log2FC, Log2 fold change. **F** Pathway Enrichment Analysis. The horizontal axis shows -Log10(FDR), reflecting the adjusted p-value; a larger -Log10(FDR) value indicates a smaller p-value. The vertical axis displays enriched pathways in three Gene Ontology (GO) terms: Biological Process (BP), Cellular Component (CC), and Molecular Function (MF). The size of the dots reflects gene counts, and their colour (FE) represents the Fold Enrichment. **G** Hub genes and their relative expression in each comparison Hub gene is defined by highest Maximal Clique Centrality (MCC) score. The horizontal axis shows -log(FDR), while the colour indicates relative up- or down-regulation of expression. A Log2FC > 0 indicates up-regulation in the “second” morphology, such as an increase in Spindle in the “NST vs. Spindle” comparison, and vice versa.

The annotation of case 1 is shown in Fig. 2A, with morphology images displayed in Fig. 2B; ROIs were integrated for analysis. In case one, 213 spatially barcoded dots were classified as NST, 1080 as spindle, and 636 as pleomorphic cells. A clustering plot of UMAPs is shown (Fig. 2C) with capture spots coloured based on manual annotation. Each morphology forms a distinct cluster, and the pleomorphic carcinomatous (epithelioid) and spindle components appear more closely related on UMAPs than NST. Notably, the spindle morphology was positioned between the NST and pleomorphic on the clustering plot (Fig. 2C), suggesting it may represent a transitional state between these two morphologies. *Monocle3* trajectory analysis demonstrated putative evolutionary paths. If we consider that NST is the starting point in MpBC, in this analysis there is transition to spindle, and subsequently from spindle to pleomorphic (Fig. 2D). Interestingly, the spindle morphology, with its looser distribution of data points and similar cell type composition across spots, suggests a more dynamic or transitional cellular state. This contrasts with the NST and pleomorphic, which demonstrate tighter clustering, indicative of more stable states. While the clusters predominantly maintained distinction, minor commonalities were observed between NST and spindle, as well as between spindle and pleomorphic (Fig. 2D). Differential expression analysis revealed 454 genes with increased expression in NST, and 211 with higher expression in spindle (Fig. 2E, Supplementary Tables 8–10). For example, spindle cells showed reduced expression of genes like *AZGP1, WFDC2* and *ERBB2*, and an increase in *VIM*, *TIMP3*, and *POSTN*. Most DEGs were noted between NST and pleomorphic, as predicted from the UMAP projection. Between NST and pleomorphic, 401 genes were higher in pleomorphic, while 557 were elevated in NST (Fig. 2E). Transcripts *CTSK, ACP5*, and *MMP13* showed reduced expression in NST, while *AZGP1, WFDC2, ERBB2*, and *SLPI* were also reduced in pleomorphic. The comparison between spindle and pleomorphic identified 176 genes as high in pleomorphic (e.g., *CTSK, ACP5, SPP1*, and *CD68)*, and 160 more highly expressed in spindle cells (e.g., *NGFR, KRT16, COL11A1*, and *AZGP1;* Fig. 2E). *AZGP1* was most highly expressed in NST, then reduced in spindle cells, with the lowest expression in pleomorphic, consistent with a progressive downregulation to the more mesenchymal/invasive feature^13^. This gradual decrease was also observed in genes such as *WFDC2, ERBB2*, and *SLPI*.

Comparing NST to spindle and pleomorphic, pathways related to tumour invasiveness and metastasis, such as cell adhesion (FDR = 3.86E-07, FDR = 1.01E-08) and cell migration (FDR = 5.38E-05, FDR = 2.50E-05) were significantly enriched (Fig. 2F; Supplementary Table 11–13). Between spindle and pleomorphic, 3/5 key biological pathways were associated with osteoclast formation: osteoclast differentiation (FDR = 3.17E-04), positive regulation of bone resorption (FDR = 5.44E-03) and osteoclast development (FDR = 6.88E-03). Furthermore, genes up-regulated in pleomorphic regions included those characteristically expressed by macrophages and osteoclasts, such as *CD68, CTSK, ACP5*, and *SPP1*^14,15^. SPP1 protein was expressed in both pleomorphic tumour cells and tumour associated macrophages (TAMs) (see section 3). Pathway analysis suggested that pleomorphic differentiation shares similarities with a macrophage lineage involved in the formation of multinucleated osteoclasts. However, this could be due to the presence of osteoclast-like giant cells embedded within pleomorphic regions^16^. Interestingly, *CSF1,* a key regulator of macrophage production and differentiation, and TAM recruitment^16^, was progressively upregulated from NST through spindle to pleomorphic regions, consistent with the variable distribution of TAMs throughout these regions histologically.

String and Cytoscape were used to infer protein-protein interaction networks (PPI) from the DEG list. The top 10 genes exhibiting the highest connectivity (hub genes), and the interaction networks of hub genes and their expanded subnetworks are shown in Fig. 2G and Supplementary Fig. 7. The NST and spindle comparison shows downregulation of *CDH1*, claudins and tight junction-related genes in NST, correlating with EMT activation^17,18^. The hub genes in NST vs pleomorphic are notably different, with several integrin family members identified, indicating interactions with the extracellular matrix. *COL4A1* and *COL6A1* are upregulated in NST and are essential collagens for connective tissue architecture^19^. A decreased LAMA4:LAMA5 expression ratio has been associated with anti-tumour immunity^20^ and in pleomorphic, a higher *LAMA4:LAMA5* ratio was observed (Supplementary Table 9), indicative of an immunosuppressive environment that may facilitate tumour immune evasion. Conversely, in NST, a lower *LAMA4:LAMA5* ratio supports an environment that activates the immune response. Case One showed moderate PD-L1 tumour cell expression, negligible FOXP3 expression, and a moderate presence of TILs (20%), suggesting that the tumour microenvironment is unlikely to be immunosuppressive^21^.

For the spindle and pleomorphic comparison, the hub genes corroborate the results from pathway enrichment analysis (Fig. 2F) where pleomorphic is associated with osteoclast development. *ACP5*^22^ and *CTSK* contribute to bone remodelling, while *CSF1* and *CSF1R* govern macrophage formation^23^. *TNFSF11* and *NFATC1* are fundamental for osteoclast and lymphocyte differentiation^24^. The ATP synthase genes^25^ are upregulated in pleomorphic relative to the spindle, indicating heightened energy requirements, likely due to enhanced cell division; this case was highly proliferative, particularly apparent in the pleomorphic areas. This case demonstrates the spindle transition phenotype during the evolution to pleomorphism.

**Fig. 3:**
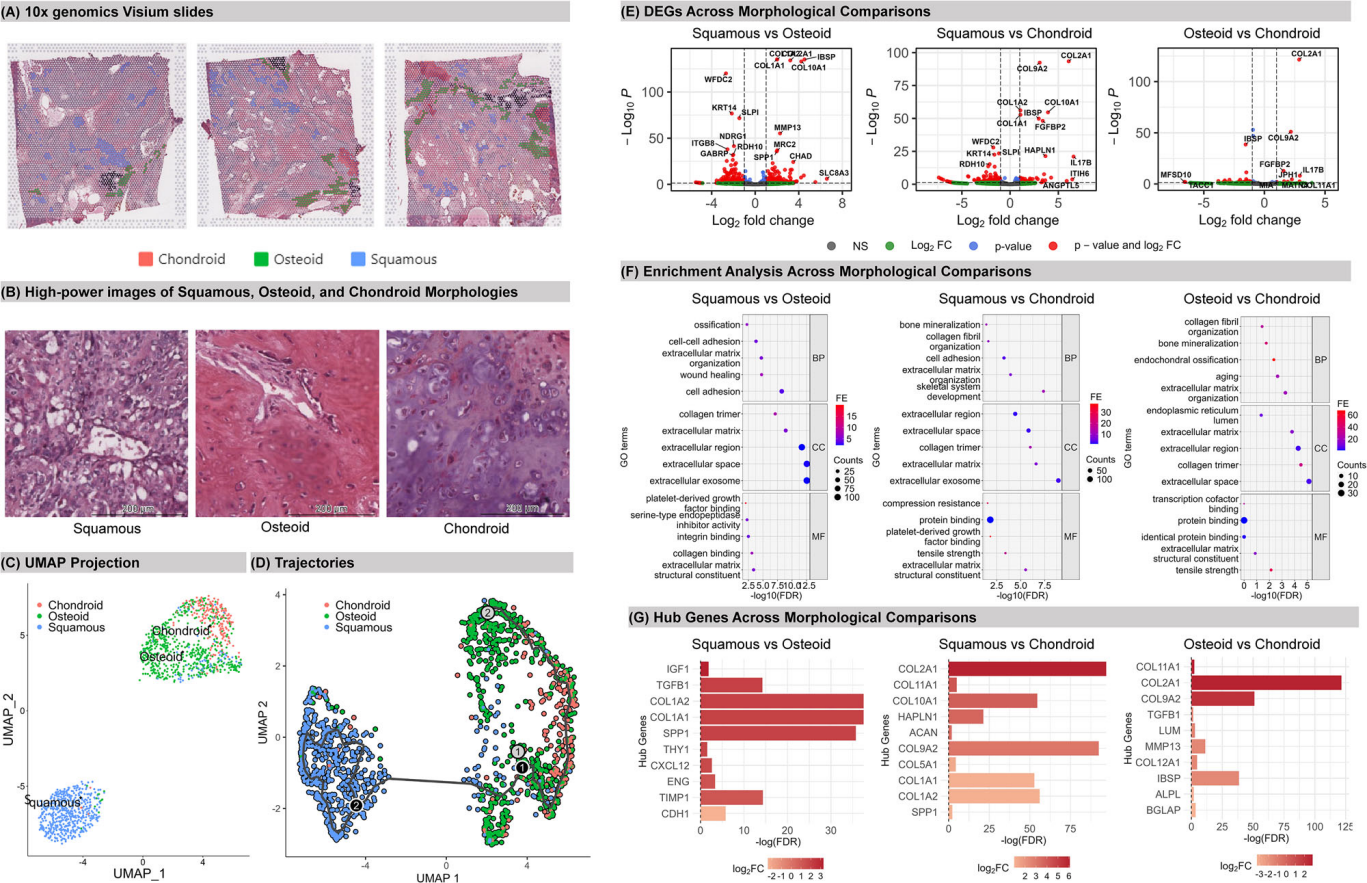
Spatial Transcriptomics Analysis of Case Two. **A** Manually annotated distinctive morphologies: squamous, osteoid, and chondroid. The squamous component was characterised by clusters of enlarged tumour cells with abundant dense eosinophilic cytoplasm and areas of keratinisation. The chondroid component contained cartilaginous stromal matrix, while the osteoid component showed overt osteoid formation. **B** High powered images of phenotypes. **C** Clustering and **D** trajectory analysis plot. Red indicates chondroid; green represents osteoid; blue denotes squamous. **E** Volcano plots for differentially expressed genes (DEGs) across three comparisons. The threshold for significant DEGs is adjusted p-value < 0.05 and |Log2FC | >1. Significant DEGs are marked in red; DEGs with adjusted p-value < 0.05 and |Log2FC | <1 are in blue; DEGs with adjusted p-value > 0.05 and |Log2FC | >1 are in green; NS, non-significant. Log2FC, Log2 fold change. **F** Pathway Enrichment Analysis. The horizontal axis shows -Log10(FDR), reflecting the adjusted p-value. The vertical axis displays enriched pathways in three GO terms: Biological Process (BP), Cellular Component (CC), and Molecular Function (MF). The size of the dots reflects gene counts, and their colour (FE) represents the Fold Enrichment. **G** Hub genes and their relative expression in each comparison. The horizontal axis shows p-values, while the colour indicates relative up- or down-regulation of expression. A Log2FC > 0 indicates up-regulation in the “second” morphology, such as an increase in osteoid in the “Squamous vs. Osteoid” comparison.

Squamous, osteoid and chondroid morphologies in Case two are shown in Fig. 3A, B. For Case two, 577 spatially barcoded dots had squamous phenotype, 569 were osteoid, and 198 were chondroid. In both the UMAP clustering plot (Fig. 3C) and trajectory plot (Fig. 3D), squamous clusters distinctly from the osteoid and chondroid clusters. Osteoid and chondroid clusters showed proximity with some overlap, indicating their relative higher ‘relatedness’. *Monocle3* trajectory analysis (Fig. 3D) suggests that chondroid may represent an evolutionary branch from the squamous cluster, however the similarity between chondroid and osteoid (overlapping distribution) might challenge the discriminatory power of the algorithm. Additionally, the distance between the squamous and the osteoid/chondroid clusters suggests there may be an intermediate cell state that was not captured in the spatial transcriptomic profiling. The pathology report notes an NST component, but this was not present in this sample block.

Statistically significant DEGs were identified, with squamous exhibiting the most differences compared to the osteoid and chondroid (Fig. 3E; Supplementary Table 14–16): 262 genes were downregulated while 197 genes were upregulated in osteoid v squamous. Between the squamous and chondroid clusters, there were 44 genes upregulated, and 146 genes that were downregulated in chondroid. Predictably, collagen-related genes were significantly overexpressed in chondroid. When comparing osteoid and chondroid clusters, just 10 genes were upregulated, and 30 genes were downregulated. As expected, squamous expressed higher levels of established markers *KRT14* and *ESRP1*^26,27^.

The DEGs correlate to pathways associated with cell adhesion, regulation of the extracellular environment, bone development, and collagen secretion (Fig. 3F, Supplementary Table 17–19). Between squamous and osteoid regions, both cell adhesion (FDR = 5.85E-09) and wound healing (FDR = 1.71E-05) pathways are significantly enriched. Between osteoid and chondroid, the enriched pathways are predictably related to bone-like differentiation; ECM organization is enriched in chondroid-overexpressed genes, while bone mineralization is enriched in osteoid-overexpressed genes. Key hub genes distinguishing osteoid from squamous (Fig. 3G, Supplementary Fig. 8) show *CDH1* is elevated in squamous and *COL1A1, COL1A2, SPP1, TGFB1, TIMP1, ENG, CXCL12, THY1*, and *IGF1* are upregulated in osteoid, as expected.

Collagen-related genes were found to be differentially expressed between osteoid and chondroid (Fig. 3G), indicating different cell fates for bone-like differentiation in MpBC. Among these hub genes*, COL2A1* is chondrogenic neoplasm marker, but *COL9A2* and *COL11A1* have not been well studied^28^. Genes like *ALPL* and *BGLAP* encode osteogenic markers, while MMPs are markers of osteosarcoma^29^. *MMP13* can activate osteoclast formation during breast cancer bone metastases, while *TGFB1* is found at high levels in osteosarcoma^29^. Lumican (*LUM*) is a basic proteoglycan bone matrix component^30^ that acts as a marker of mature osteoblasts and is highly expressed within the newly-formed bone matrix after bone mineralization. Muscle-derived Lumican can stimulate bone formation through the actions of integrin α2β1 and ERK signalling^30^. This suggests chondroid cells have the capacity to differentiate into osteoid cells in MpBC. In addition, bone sialoprotein (*IBSP*), a glycoprotein in bone tissue, is highly expressed in mature bone cells instead of in their immature precursors^31^. *IBSP* expression showed a significant increase from squamous to chondroid, then to osteoid, indicating that *IBSP* expression may represent the final stage of bone-like differentiation in MpBC. Between squamous and chondroid, all 10 hub genes were upregulated in chondroid compared with squamous; predictably, most of these were collagen-associated genes.

**Fig. 4:**
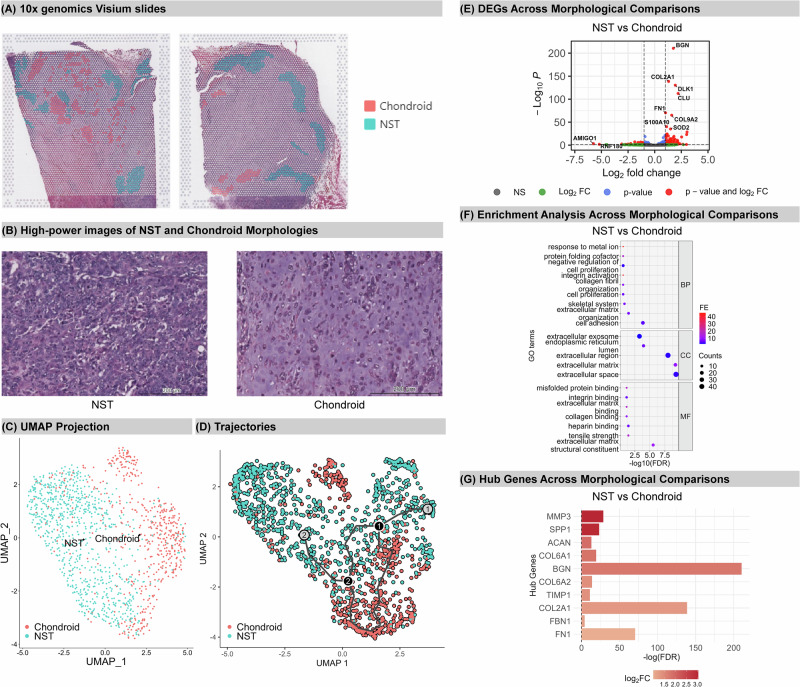
Spatial Transcriptomics Analysis of Case Three. **A** Manually annotated distinctive morphologies: chondroid and NST. **B** High powered images of phenotypes. **C** Plot for clustering and **D** trajectory analysis. Red represents chondroid; blue indicates spindle. **E** Volcano plot for differentially expressed genes (DEGs) across three comparisons. The threshold for significant DEGs is adjusted p-value < 0.05 and |Log2FC | >1. Significant DEGs are marked in red; DEGs with adjusted p-value < 0.05 and |Log2FC | <1 are in blue; DEGs with adjusted p-value > 0.05 and |Log2FC | >1 are in green; NS, non-significant. Log2FC, Log2 fold change. **F** Pathway Enrichment Analysis. The horizontal axis shows -Log10(FDR), reflecting the adjusted p-value. The vertical axis displays enriched pathways in three GO terms: Biological Process (BP), Cellular Component (CC), and Molecular Function (MF). The size of the dots reflects gene counts, and their colour (FE) represents the Fold Enrichment. **G** Hub gene and relative expression between chondroid and NST. The horizontal axis represents p-values; colour indicates relative up- or downregulation. All hub genes with Log2FC < 0 are down-regulated in the comparison of chondroid to NST.

In case three, NST and chondroid elements were studied (Fig. 4A, B), while spindle and rhabdoid morphologies noted in the pathology report were not identified in the examined sections. In the cluster analysis, the manually annotated NST (834 spatially barcoded dots) and chondroid spots (519 spatially barcoded dots) were distinctly segregated (Fig. 4C). However, in the trajectory analysis, the estimated cell fate pathway formed a circular pattern (Fig. 4D). *Monocle3* is commonly used for data types with multiple discrete clusters, where the transition between clusters is expected to be continuous along the manifold; therefore, this approach presents challenges in effectively predicting differentiation between two distinct morphologies^32^. In fact, there was extensive transcriptomic similarity between these regions, with few DEGs identified (Fig. 4E, Supplementary Table 20). In NST, 53 genes were upregulated compared to chondroid, (e.g., *LGR5)* while 98 genes showed higher expression in chondroid. Expectedly, the significantly enriched pathways predominantly associated with extracellular matrix (ECM) (Fig. 4F, Supplementary Table 21). All identified hub genes were overexpressed in chondroid relative to NST (Fig. 4G, Supplementary Fig. 9). Among these hub genes, *FN1* is a frequent target of translocation, resulting in fusions with tyrosine kinase genes in cartilaginous soft tissue neoplasms^33^ and *MMP3*, stromolysin, modifies the ECM, and promotes EMT and metastasis^34^.

In case three, chondroid exhibits active collagen formation and bone remodelling like case two. Combined with the fact that many enriched pathways were related to the extracellular matrix, this suggests that NST in case three may be starting to secrete cartilage-specific collagens (such as type II collagen) and other ECM components like aggrecan (*ACAN*), leading to a transformation into cartilage-like cells.

### Protein expression recapitulates spatial transcriptomics

**Fig. 5:**
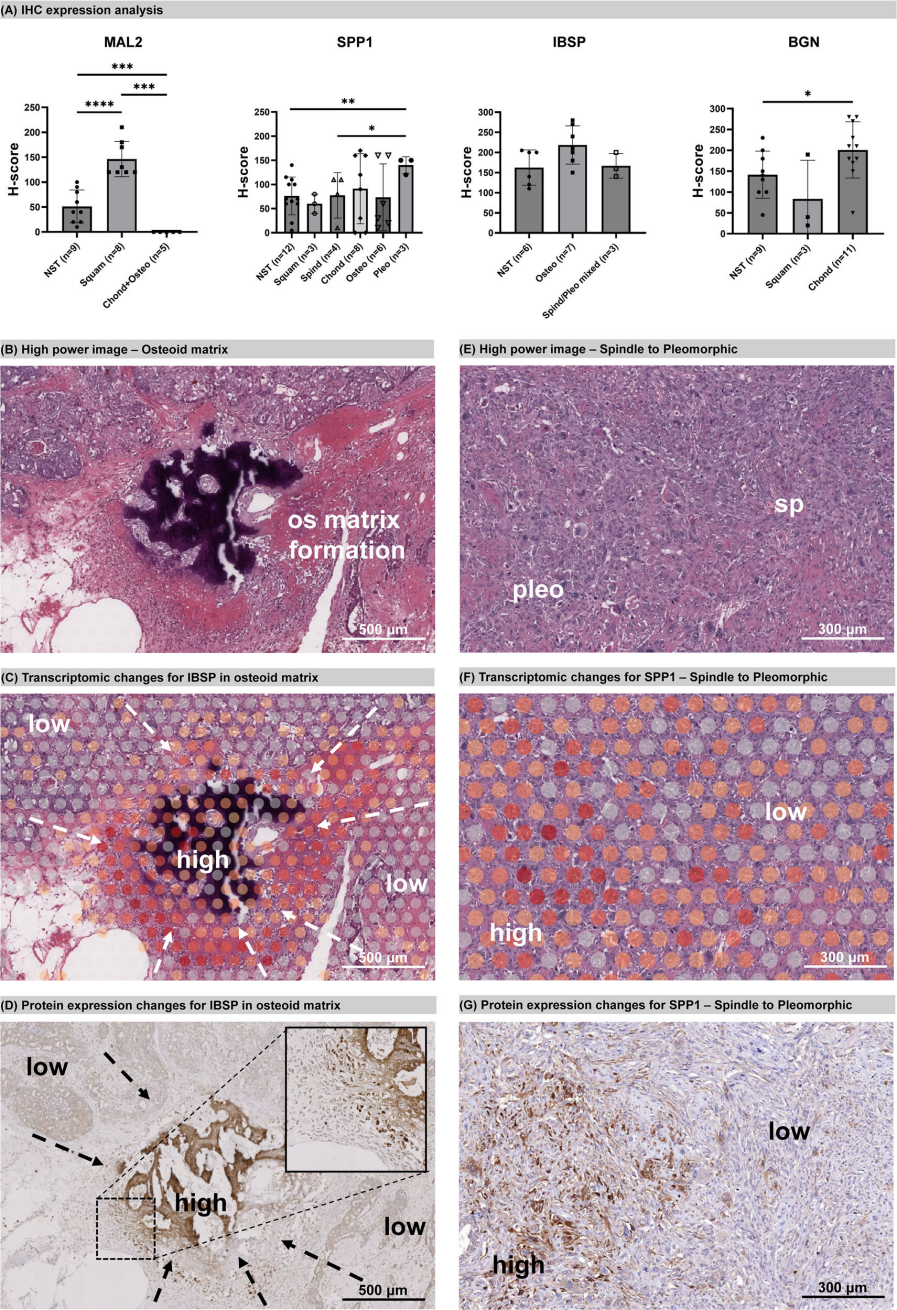
Immunohistochemistry validation of candidates. **A** Protein expression for MAL2, SPP1, IBSP, and BGN was evaluated by IHC H-score (mean with SD). Asterisks indicate statistical significance: *p ≤ 0.05; **p ≤ 0.01; ***p ≤ 0.001; ****p ≤ 0.0001. The x-axis indicates phenotype and sample size. **B** High-power image of the osteoid matrix in case two. **C** Transcriptomic changes for *IBSP* in the osteoid matrix, with arrows indicating expression levels from low to high. **D** IHC for IBSP shows protein expression changes in the osteoid matrix, with arrows indicating expression levels from low to high. **B**–**D** show the same region. **E** High-power image of the spindle to pleomorphic transition region in case one. **F** Transcriptomic changes for *SPP1* from spindle to pleomorphic, with arrows indicating expression levels from low to high. **G** IHC for SPP1 shows expression changes from low (spindle) to high (pleomorphic). **E**–**G** show the same region.

We selected four hub genes with high expression in specific morphologies for immunohistochemistry (IHC) validation in a panel of MpBC (Supplementary Table 1, Supplementary Fig. 10) on the basis of their significant *P* value, high fold change and literature context. *MAL2* was highly expressed in squamous cells, and *IBSP* was highly expressed in osteoid cells. *SPP1* was upregulated in pleomorphic, while *BGN* was highly expressed in chondroid cells in Case three. MAL2, SPP1, and BGN had significantly higher H-scores compared to other morphologies, consistent with spatial transcriptomics results (Fig. 5A). Although IBSP did not show a significantly higher H-score in osteoid than other phenotypes, it showed strong (3 + ) staining in the calcified osteoid matrix in all samples (*n* = 7) and a clear increase in expression during the formation of the calcified osteoid matrix within the tumour (Fig. 5B–D). This gradient of change was consistent at both the transcriptomic level from Visium profiling (Fig. 5C) and the protein level (Fig. 5D). Similarly, *SPP1* expression correlated with the morphological change from spindle to pleomorphic (Fig. 5E, F), also observed at the protein level (Fig. 5G). In addition to staining pleomorphic tumour cells, SPP1 predictably showed strong staining in macrophages (including multinucleated giant cells) and other inflammatory cells (excluded from analysis), as well as malignant osteoid (basophilic/calcified osteoid).

### Integrated Analysis of transcriptomics and methylation profiling identifies common pathways and transcripts potentially driving intra-tumoral heterogeneity

Three integrative cross-analyses were undertaken between spatial transcriptomic and methylation datasets (Supplementary Table 22). DEGs and genes with significant MVPs were integrated to identify those genes with both differentially expressed and multiple differentially methylated CpGs (Supplementary Table 23). Gene Ontology analysis (Supplementary Table 24) as well as STRING and Cytoscape were used to identify the top integrated hub genes (Supplementary Fig. 11). In the NST versus spindle comparison, common pathways were identified in cellular interaction, movement, and tissue formation (Supplementary Table 24) and hub genes were *ERBB2, ITGB3, ITGB4, VIM*, and *JUP*. Comparing the Visium-derived expression of these hub genes across the different morphologies in the context of their methylation status (Supplementary Fig. 12) showed concordance between methylation status and expression level for *ERBB2, ITGB3*, and *ITGB4*. In the NST vs. chondroid analysis, common pathways involved cellular attachment, tissue structure maintenance, cell growth control, and collagen organization (Supplementary Table 24); the top 5 hub genes were *FN1, COL6A1, COL6A2, COL2A1* and *COL16A1* and all were concordant between transcriptomic and methylation analyses (Supplementary Fig. 13). For the squamous vs. chondroid comparison, the common pathways involved skeletal system development and regulation, focusing on cell adhesion, collagen structure, and control of cell growth and gene transcription (Supplementary Table 23). The top 5 hub genes were *COL2A1, COL1A1, COL1A2, ITGB4* and *ITGB8*, of which only the collagens displayed concordance for the transcriptomic and methylation profiles (Supplementary Fig. 14).

We then validated these hub genes using an independent MpBC dataset (GSE212245), which applied the NanoString Breast Cancer 360 (BC360) Panel of 770 genes in 27 MpBC cases^9^. Hub genes *ERBB2, ITGB3, VIM*, and *COL2A1* are included in the BC360 panel and their expression levels across various metaplastic components was assessed (Supplementary Fig. 15). All four transcripts showed consistent results with our transcriptomic profiling, however *ERBB2* and *ITGB3* did not reach significance. *ERBB2* in NST, *ITGB3* and *VIM* in spindle, and *COL2A1* in chondroid, all showed higher median values compared to other metaplastic components. Four significant relationships were identified using one-way ANOVA: *VIM* expressed in spindle was significantly higher than in squamous (*p* = 0.0122), and *COL2A1* in chondroid was significantly higher than in NST (*p* = 0.0006), spindle (*p* = 0.0159), and squamous (*p* = 0.0208).

### Transcription Factor Enrichment Analysis

**Fig. 6:**
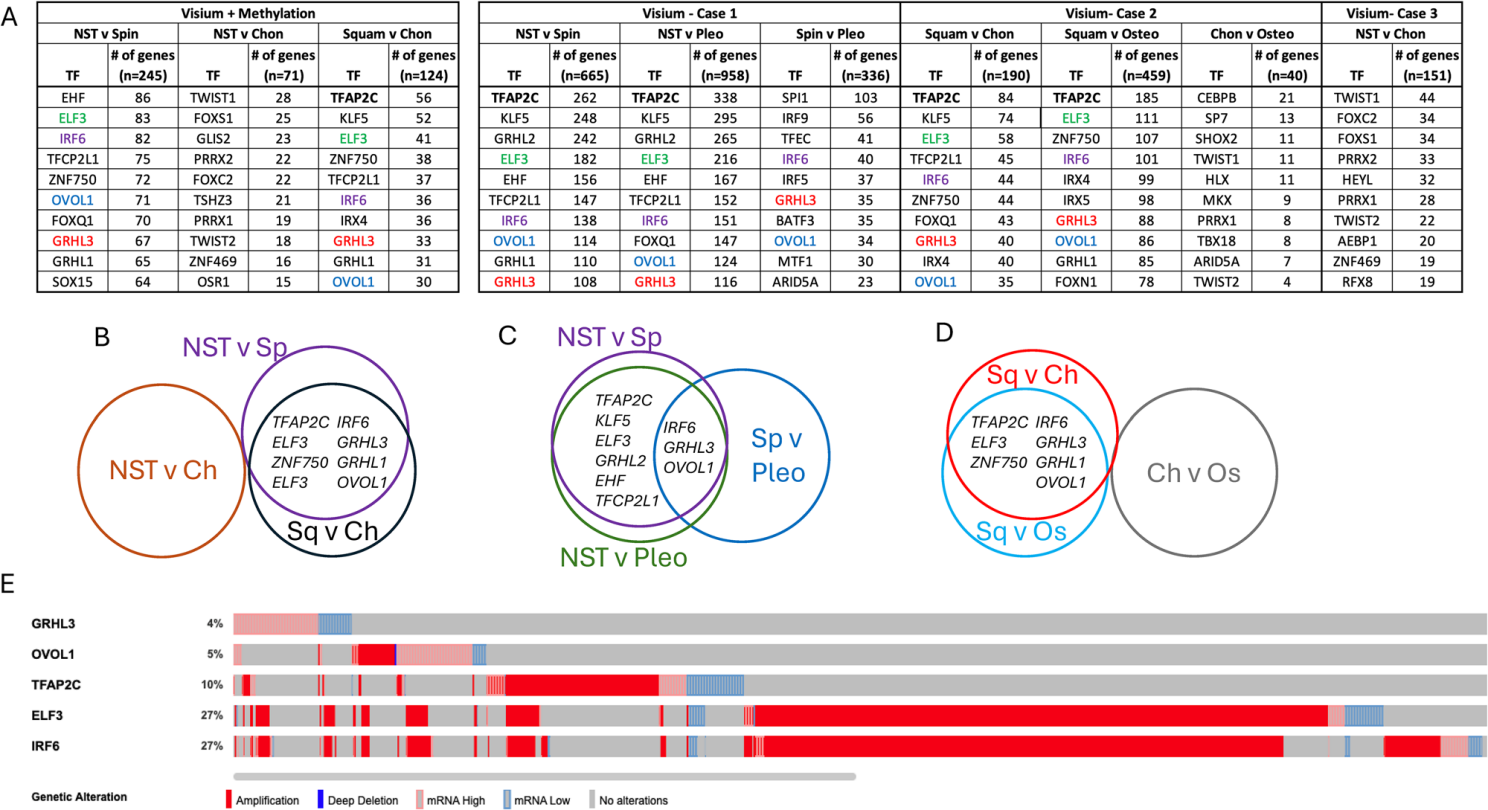
Master transcription factors are enriched across metaplastic morphology comparisons. Using the ChEA3 algorithm, differently expressed genes were examined for TF binding sites; the number of input DEGs is noted for each comparison. The top ten TFs for each DEG list are shown in (**A**), with the number genes featuring the sites also noted for the integrated methylation and DEG, and the Visium DEG lists alone. **B**–**D** highlight common TFs across the comparisons. **E** Oncoprint of METABRIC breast cancer data, highlighting genomic alterations and their prevalence in *GRHL3, TFAP2C, OVOL1, ELF3* and *IRF6*. Invasive carcinoma-no special type, NST; spindle, spin or Sp; squamous, squam or Sq; chondroid, chond or Ch; osteoid, osteo or Os.

Finally, we investigated whether a master transcription factor/s (TFs) may account for the morphology-specific differential gene expression. Both the integrated gene lists and the DEGs from Visium analyses were examined using ChEA3^35^, a TF target over-representation analysis tool. The identified TFs were ranked based on the statistical significance of the overlap between input gene sets and the ChEA3 TF target genesets. The top 10 TFs for all comparisons are shown in Fig. 6, and the similarities of these gene lists are shown by the weighted Venn diagrams. Well-characterised drivers of EMT such as *TWIST1, TWIST2* and *FOXC2* were only present in comparisons with chondroid or osteoid morphologies^2^. TGF-β, an EMT inducer, was up-regulated in pleomorphic compared to NST in case one, and up-regulated in osteoid compared to chondroid and squamous in case two. Across the other comparisons, several TFs were repeatedly identified: *GRHL3* (7 comparisons), *OVOL1* (7 comparisons), *IRF6* (7 comparisons), *ELF3* (6 comparisons), and *TFAP2C* (5 comparisons). A range of transcription factors linked to mesenchymal-epithelial transition (MET) were also identified, including *GRHL1, GRHL2* and *KLF5*^36^; which engage in mutually inhibitory interactions with transcription factors that induce EMT^36^.

*GRHL3* encodes a member of the Grainyhead TF family. Functionally, in addition to embryonic development roles including epithelial lineage commitment^37^, GRHL3 also induces multinucleation, and suppresses SCC in mice^38^. In BC, low levels of GRHL3 associate with high grade tumours and TNBC, however pro-tumorigenic relationships have also been noted^39^. Here, *GRHL3* expression increased progressively from NST to spindle (Log2FC = 2.32, *p* = 0.0059) and then to pleomorphic (Log2FC = 2.54, *p* = 0.011), suggesting a potential role in the formation of pleomorphic cells. Notably, *TFAP2C* was identified in 5 comparisons, with the highest number of target genes. In case two, *TFAP2C* was up-regulated in squamous compared to osteoid (log2FC = 2.17, *p* = 3.28E-4). *TFAP2C*/AP2γ regulates pluripotency by opening enhancers to maintain an embryonic stem cell state and its high levels are a marker of poor prognosis in BC^40^. Ovo-like transcriptional repressor 1 (*OVOL1*) maintains the epithelial identity of BC cells by inhibiting EMT through the degradation of the TGF-β type I receptor^41^. Similarly, *IRF6* (Interferon regulatory factor 6), a key regulator of epithelial adhesion and has non-TF roles in the recycling of E-cadherin, is also a favourable prognostic marker in BC, with high levels enhancing treatment sensitivity and inhibiting tumorigenesis^42^. In cancer, hypermethylation of *IRF6* is implicated in the progression from vulvar lichen sclerosis to vulvar squamous cell carcinoma^43^. Notably, both neurulation and epidermal differentiation are regulated by the TFAP2A-IRF6-GRHL3 genetic pathway^44^.

*ELF3* is associated with epithelial differentiation and homeostasis^36^. Consistently, we show that *ELF3* expression is higher in NST compared to pleomorphic (Log2FC = 4.41, *p* = 1.18E-21) and spindle (Log2FC = 3.70, *p* = 2.99E-31) in Case one. Similarly, *ELF3* shows higher expression in squamous relative to osteoid (Log2FC = 1.69, *p* = 2.13E-4) and chondroid (Log2FC = 2.66, *p* = 2.75E-3) in Case two. Among these frequently identified TFs, only *ELF3* and *IRF6* are present in the BC360 Panel data (GSE212245)^9^. Supplementary Fig. 16 shows that the expression level of *ELF3* in NST and squamous had a higher median value than spindle (NST vs. spindle, *p* = 0.024, one-way ANOVA) and chondroid, consistent with our data. For *IRF6*, expression in chondroid (p = 0.019) and squamous (*p* = 0.0157) was significantly higher than in spindle. Our attempts to further validate in an independent metaplastic dataset were hampered by a significant rate of probe dropout in the Weigelt set (*n* = 28; GSE57544)^45^.

To determine whether these TFs are altered in BC more broadly, we used the METABRIC data^46^. The oncoprint in Fig. 6E emphasises a high frequency of amplification for *ELF3* and *IRF6* (27%). High mRNA levels are shown for *GRHL3* and *OVOL1*, but in a smaller proportion of cases (4% and 5%, respectively), while *TFAP2C* is altered in 10% of cases. Notably, none of these genes are considered to be BC driver genes^47^, however the frequencies of alteration indicate potential associations with BC. Contingency analysis in METABRIC (Supplementary Table 25) showed that genomically altered *GRLH3* was associated with the claudin-low phenotype (*p* = 8.401e-5), Integrative cluster 10 (*p* = 2.532e-3), triple negativity (‘3-gene classifier’; *p* = 3.137e-5) and grade 3 (*p* = 6.567e-3): all broadly ‘metaplastic carcinoma’ features. Similarly, altered *TFAP2C* was also significantly enriched in ER-negative cases, but was additionally associated with luminal B as well as claudin-low cancers. Alterations in both *ELF3* and *IRF6* were significantly enriched in luminal A (typically ER-positive) cancers. Supplementary Figs. 17 and 18 show detailed analysis of the METABRIC cohort, assessing the expression of the TFs by quartile, and considering ER status in terms of expression level and survival impact. Each TF showed a significant difference in mRNA level between ER-negative and -positive cases, with highest expression levels in ER-negative for all excluding *OVOL1*. Indeed, *OVOL1* expression was also significantly enriched in luminal A and B subtypes (Supplementary Fig. 17J), while *TFAP2C, GRHL3, IRF6* and *ELF3* showed significant enrichment in Basal and HER2 subtypes (Supplementary Fig. 17E, O; Supplementary Fig. 18E, J). *ELF3* was statistically significant in KM analyses, with high expression associating with the poorest outcomes (Supplementary Fig. 18G–I). High levels of *TFAP2C* showed a clear split of curves in ER-negative cases, with higher expression associated with better survival in ER-positive cohort (Supplementary Fig. 17D). KM-Plotter^48^ analysis of RNASeq data restricted to high-grade, basal BCs (most metaplastic-like) showed significant association between high expression and poorer survival for *OVOL1* and *TFAP2C* (Supplementary Fig. 19), however *IRF6* and *ELF3* showed the converse, with lower expression associating with poorer survival.

## Discussion

Metaplastic breast cancer (MpBC) is the archetypal ‘stem cell’ tumour, with its diverse morphologies and vast intra-tumoral heterogeneity. Mixed MpBC constitute the majority (>70%) of all metaplastic BCs and comprise carcinoma of No-Special Type (NST) with combinations of heterologous elements like squamous, spindle, chondroid, osteoid, and rhabdoid within the one tumour^1^. While the different metaplastic components are clonally related at the genetic level^8^, the expression profile of NST reflects the matched heterologous element^9^. This data, combined with ours, implies that the molecular nature of the NST components determines the pattern of lineage differentiation, and that the NST is the default origin morphology as in other heterogeneous BCs^49^. Conceivably, if commitment to lineage occurs early, it results in NST loss and complete conversion of phenotype; or, if later in the tumour’s evolution, results in partial conversion, and persistent NST. Early commitment to phenotype could account for the pure spindle and pure squamous MpBC presentations. It remains to be seen whether those metaplastic cases without identifiable NST components have fully committed to a new lineage or whether the multipotent progenitor theory applies. Indeed, it is possible that both these evolutionary pathways may exist.

In addition to novel findings, our analyses reproduced established correlations: squamous and NST morphologies show higher expression of epithelial markers such as E-cadherin and claudin, while spindle, chondroid and osteoid predominantly express mesenchymal-related markers, including *SPP1, COL1A1*, and *COL1A2*^50^. Analysis of differently methylated regions showed that squamous components of MpBC are more like normal squamous cells than other squamoid malignancies. This increased similarity to normal squamous cells may explain why the pure squamous subtype of MpBC (without other features) shows a more favourable prognosis than other MpBCs^5^. Conversely, the chondroid regions were more like malignant entities such as chondrosarcomas, where a series of biomarkers are typically hypomethylated^12^. Methylation status infers *IL6R* is likely up-regulated in chondroid which aligns with previous findings that IL6R/IL6 promotes chondrogenic differentiation in human mesenchymal stem cells^51^. Indeed, high levels of IL6R/IL6 are correlated with reduced survival and treatment resistance in BC^52^. In chondroid regions, *OSBPL5* was 5’UTR hypermethylated and hypomethylated in the gene body, suggesting that *OSBPL5* is suppressed in chondroid compared to spindle, and similarly germline variations in *OSBPL5* 3’UTR associate with poor outcomes in BC^53^.

Spindle and chondroid showed more hypermethylated CpGs compared to NST and squamous, and the majority of which were located on the gene body. *SHANK2* and *MAD1L1* were among the top 5 differentially methylated genes in all epithelial versus mesenchymal comparisons. *SHANK2* has not been well studied in BC, but was shown to induce drug resistance in advanced oropharyngeal carcinomas^54^, and high levels of *MAD1L1* expression correlate with reduced tumour cell sensitivity to chemotherapy^55^. It remains to be seen whether there are similar clinical relationships in BC. In addition, *PTPRN2* and *HDAC4* showed a higher number of significantly methylated CpG sites compared to other genes. The high proportion of hypomethylated CpGs within the gene bodies in the epithelial group suggests that *PTPRN2* and *HDAC4* may be downregulated in NST and squamous cells compared to spindle and chondroid. This is consistent with previous literature, as PTPRN2 (Protein Tyrosine Phosphatase Receptor Type N2) is related to insulin secretion and has been shown to facilitate tumour metastasis by reducing plasma membrane PI(4,5)P₂ levels, and is therefore upregulated in highly metastatic breast cancer cells^56^. The role of HDAC4 (Histone Deacetylase 4) in breast cancer remains unclear, as its function is context dependent, whether promoting or suppressing tumour growth^57^. In our study, HDAC4 seems to be upregulated in mesenchymal metaplastic components in MpBC.

High-resolution trajectory analysis showed that spindle cells may in fact be a transitional state between NST and pleomorphic morphology. Our data support the idea that pleomorphic carcinomas represent endpoint dedifferentiation of NST or metaplastic spindle cell carcinoma^1^. p120 catenin (*CTNND1*) loss is associated with an epithelial cell shift into a spindle/pleomorphic phenotype^58^; this is also supported here, where *CTNND1* was downregulated in spindle (LogFC = -0.957, FDR = 0.001) and pleomorphic cells (LogFC = -0.935, FDR = 0.004) compared to NST. High cellular pleomorphism correlates with a poorer prognosis, and our gene expression analysis reveals that pleomorphic regions harbor significantly higher expression of *ACAN, CTSK, CSF1*, and *CSF1R*, which are associated with poor prognosis^59^. Our spatial transcriptomics data, corroborated with IHC, also demonstrated an increase in normal bone development markers like IBSP from chondroid to osteoid morphology. This suggests that osteoid features may represent a terminal stage of progression for some chondroid regions in MpBC, potentially a form of endochondral ossification.

Upregulation of hub gene *SPP1*, and its corresponding protein in both pleomorphic tumour cells and TAMs (predominantly comprising osteoclast-like giant cells) was shown for Case One. This is likely related to the high concentration of TAMs in the tumour microenvironment of the pleomorphic component and may have prognostic significance. Indeed, recent reports have implicated CTSK, ACP5 and SPP1/osteopontin in facilitating EMT and promoting metastasis in a number of cancers – particularly those with a high concentration of CD68-positive M2-like TAMs, which play a key role in invasion, metastatic spread and drug resistance^37,38^ e.g. in lung adenocarcinoma^60^. In BC, *SPP1* expression is increased in primary tumour dormancy, and increases significantly in recurrent tumours^61^; however we show that a subset of primary metaplastic BCs has a high level of both SPP1 mRNA and protein.

Taking a broader view, we identified several TFs that may be high-level drivers of intratumor heterogeneity in MpBC; *GRHL3, ELF3*, and *TFAP2C* were upregulated in epithelial compared to mesenchymal components. Survival analysis showed high expression of *OVOL1* and *TFAP2C* was associated with poorer survival, while high *IRF6* and *ELF3* expression correlated with better prognosis.

There are several limitations associated with working with archival samples of a relatively rare subtype. Firstly, access to well-annotated samples is challenging. The high sample dropout rate for our methylation profiling was disappointing, and we were unable to access further samples of sufficient DNA quality. The nature of formalin fixation means there may be potential bias in methylation pattern analysis, affecting the number of CpGs passing QC. The Visium FFPE analysis was an early adoption of the technology, which remains a comprehensive discovery option, although this technology is not at a single cell resolution. Despite these limitations, our data reveals new insights into metaplastic BC.

In conclusion, our analyses collectively peel back another layer of complexity of metaplastic BCs, the exemplar of intratumoral heterogeneity. We have clarified our understanding in some areas and raised interesting hypotheses about differentiation pathways and associated genes. Metaplastic BCs remain an intriguing BC subtype in need of further study.

## Methods

### Samples

The sample cohort^5^ information is provided in Supplementary Table 1. All samples were archival, formalin-fixed, paraffin embedded (FFPE) tissues, and challenges around FFPE derived DNA quality, and the limitations of tissue meant not all comparisons are possible for each morphology. In total there were 29 cases: three for spatial transcriptomics; *n* = 18 samples from 17 cases for methylation profiling; *n* = 26 blocks from 18 cases for IHC validation. The research was approved by the Human Research Ethics Committees at The University of Queensland (2005/000785) and the Royal Brisbane and Women’s Hospital (2005/022).

### Tumour microdissection and DNA extraction

Tumour regions were annotated with a consultant pathologist (AS) enriching for a single phenotype where possible, with minimal tumour-infiltrating lymphocytes (TILs) to reduce normal contamination. Samples were sectioned (10 μm, 15 sections/sample), needle-dissected and DNA extracted (QIAamp DNA FFPE Tissue Kit, QIAGEN).

### Methylation profiling and analysis

Infinium FFPE DNA Restoration Solution (Illumina, US) was used for restoration of FFPE-origin DNA. 250 ng of DNA was bisulfite converted with EZ-96 DNA Methylation kit (Zymo research) and hybridised to the Illumina Human Methylation EPIC BeadChip at Australian Genome Research Facility. Samples passed quality control (QC) if >80% of the CpGs had a p-value < 0.01. Samples with less than 20% failed CpGs were recognized as having passed quality control; of 32 profiled samples, 18 samples from 17 patients passed. Failed samples were typically of poor DNA quality consistent with sample age.

The R package *ChAMP v3.18* was used for data processing^62^. QC, normalization, and batch effect correction were performed using the functions *champ.QC*, *champ.norm*, and *champ.runCombat*. Significant methylation variable positions (MVPs) with |Δβ | > 0.2, p-value < 0.01 were identified with *champ.DMP*. The genes with several significant MVPs greater than the average number in each comparison were classified as enriched genes for further analysis. Differentially methylated regions (DMRs) with a p-value < 0.01 for each comparison were identified using the Probe Lasso algorithm by running the *champ.DMR* function.

### Spatial transcriptomic profiling and analysis

The FFPE tissue blocks were processed as per manufacturer’s instructions. In brief, 5 µm tissue sections were placed on a Visium Spatial Slide (10X Genomics). Hematoxylin and eosin (H&E) staining of the tissue sections were imaged using Aperio XT Brightfield Automated Slide Scanner (Leica, DE; 20x). Sections were destained and de-crosslinked prior to hybridisation with human transcriptome probes which underwent probe extension and library construction. The libraries were sequenced at the UQ Institute for Molecular Bioscience, using Illumina NovaSeq 6000 (Illumina) with paired-end dual-indexing (28 cycles for Read 1, 10 cycles for i7, 10 cycles for i5, 50 cycles for Read 2) at a depth of ~25000 reads per section. Mitochondrial and ribosomal genes were removed before normalization. Low-quality genes (expressed in less than 3 spots/barcodes) and low-quality spots (expressing less than 10 genes) were also excluded from further analysis.

Sequencing data were demultiplexed using *bcl2fastq* software (v2.20.0.422, Illumina). The *SpaceRanger* Software v2.0.0 (10X Genomics) and the GRCh38-2020-A reference genome were used to process fastq files and map the reads to H&E images. Morphologically distinct regions of all cases were manually annotated by a pathologist (AS) using *Loupe Browser v6.0* (10X Genomics), linking the raw mapped sequencing data to morphology. The R package *Seurat v4.4.0* was used for data processing^63^. The spatial data and annotations were integrated by incorporating annotation data into the *Seurat* object metadata, aligning each annotation with its corresponding spatial spot based on ‘rownames’ in ‘meta.data’. QC and *SCTransform*-based normalization were performed. For clustering, batch effects were removed using Anchor Integration, followed by graph-based clustering using *FindNeighbors* and *FindClusters* in Seurat. Regions from the same patient were compared to identify differentially expressed genes (DEGs) between morphologies using GLM framework of *edgeR v3.19* on pseudobulked data^64^. All DEGs met two criteria: an FDR (False Discovery Rate) < 0.05 and a fold change greater than 2-fold.

The R package *Seurat v4.4.0* and *Monocle v3.0* were used for clustering and trajectory analyses^32,63,65^. Seurat object was loaded and transformed to monocle object by using *as.cell_data_set* function. Normalization involved estimating size factors, followed by dimensionality reduction. The dataset underwent alignment using Principle Component Analysis (PCA) and Latent Semantic Indexing (LSI) methods using *align_cds* function in Monocle, preparing it for nonlinear dimensionality reduction with Uniform Manifold Approximation and Projection (UMAP) using *reduce_dimension* function in *Monocle*^66^. Seurat-based annotations were integrated into the *Monocle* object for trajectory visualization.

### Integration enrichment analysis

Differentially expressed genes (DEGs) and differentially methylated genes were input into *DAVID* to identify enriched biological pathways by using the modified Fisher’s exact test. These analyses focused on three Gene Ontology (GO) terms: biological process (BP), cellular component (CC), and molecular function (MF), as well as KEGG (significance: adjusted p-value < 0.05). Transcription factor (TF) enrichment analysis was performed using ChIP-X Enrichment Analysis 3 (ChEA3), a transcription factor enrichment analysis tool that identifies TFs that are over-represented as regulating genes in the input DEG lists^35^. The top 10 TFs were selected for each comparison.

### Clinical cohort analysis

METABRIC data^46^ were accessed through cBioPortal^67^ and analysed in Prism V10. Mann Whitney U Test, Welch’s ANOVA and Log-rank tests were applied as appropriate, with P < 0.05 noted as significant.

### Protein-protein interaction networks and hub genes

Protein-protein interaction (PPI) networks were estimated using *String v12.0*. DEGs were mapped to their corresponding proteins and hub proteins and their corresponding genes (hub genes), which are highly connected to other units in the PPI network, were analysed using the *Cytohubba* v0.1 plugin in *Cytoscape* v3.9.1. The Maximal Clique Centrality (MCC) algorithm was used to rank the top 10 hub genes based on their MCC scores^68^.

### Immunohistochemistry

Immunohistochemistry was performed as standard^5^. Antigen retrieval was performed in citrate buffer (pH 6.0) for MAL2 and SPP1, and in Tris-EDTA buffer (pH 9.0) for IBSP and BGN. The slides were incubated with primary antibodies targeting MAL2 (Merck, ZRB2741, 1:100), SPP1 (Merck, HPA027541, 1:50), IBSP (Invitrogen, PA550633, 1:200), and BGN (Abcam, EPR20235, 1:5000) for 16 hours at 4°C. Staining was visualized using the MACH1 HRP detection kit (Biocare Medical). Protein expression was evaluated using an H score, calculated as the product of the proportion of stained cells and intensity (0: negative; 1: weak; 2: moderate; 3: strong). The final score for each morphology was determined by the formula: 1 × (% of 1+ cells) + 2 × (% of 2+ cells) + 3 × (% of 3+ cells).

## Supplementary information

Supplementary Tables

Supplementary Figures

## Data Availability

The profiling data supporting this study have been deposited in the GEO database (methylation: GSE278722; spatial transcriptomics: GSE283412).

## Code availability

The code for performing key analyses in this study is available at https://github.com/YF972/MpBC_EPIC_Spatial.
