# Supplementary material for: Integrated profiling of metaplastic breast cancer identifies putative master regulators of intratumoral heterogeneity
Source: NPJ Breast Cancer. 2025 Aug 11;11:89. doi: 10.1038/s41523-025-00807-x (PMC12340010; doi:10.1038/s41523-025-00807-x)
Supplement: Supplementary file 2 — Supplementary Figures [file 41523_2025_807_MOESM2_ESM.pdf]

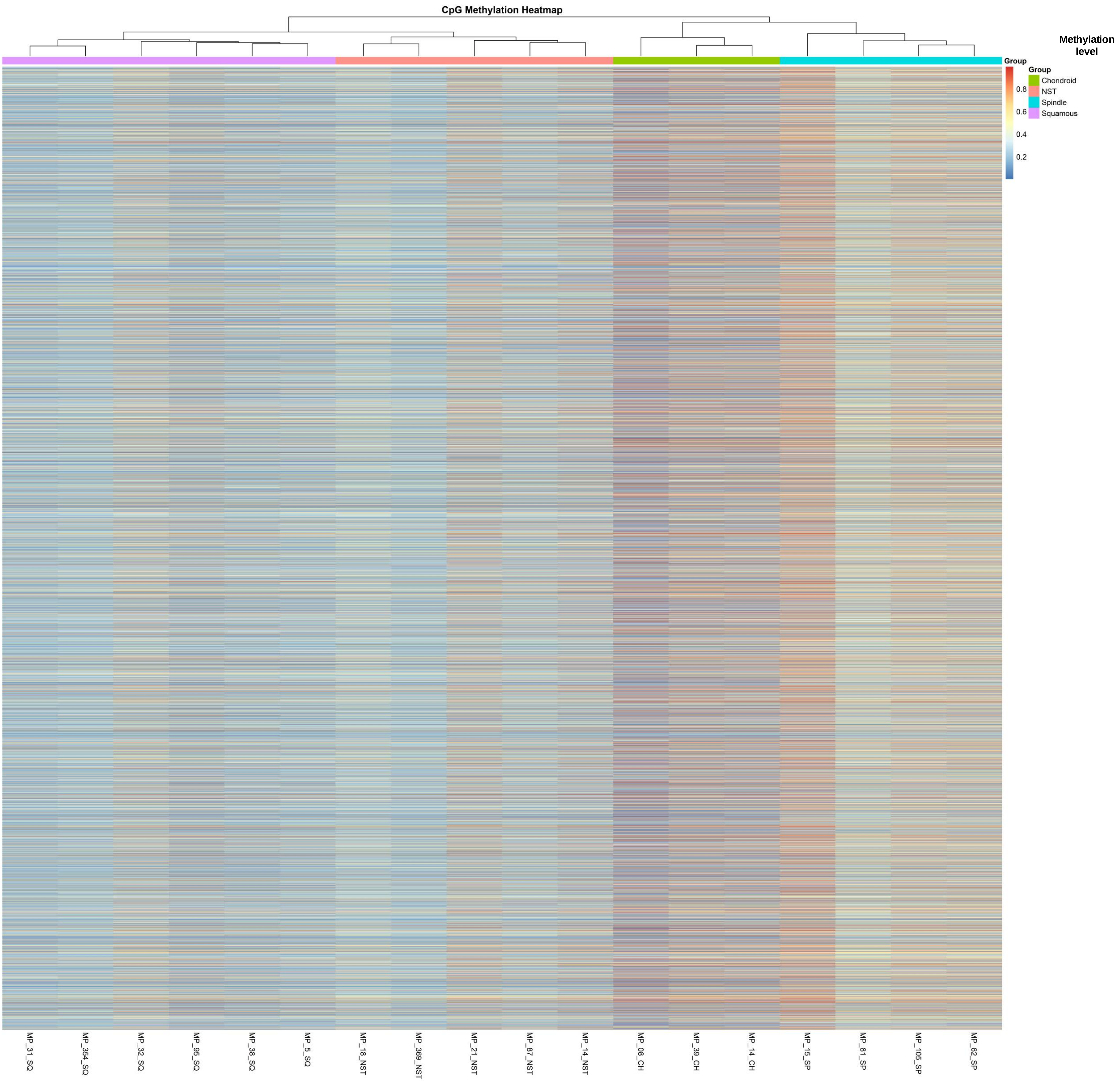

Supplementary Figure 1. Heatmap of unsupervised clustering of CpG methylation profiles across metaplastic breast cancer (MpBC) samples. The horizontal axis represents the cases, with colours indicating methylation levels ranging from 0 to 1. Red colour representing high methylation level and blue colour representing low methylation level.

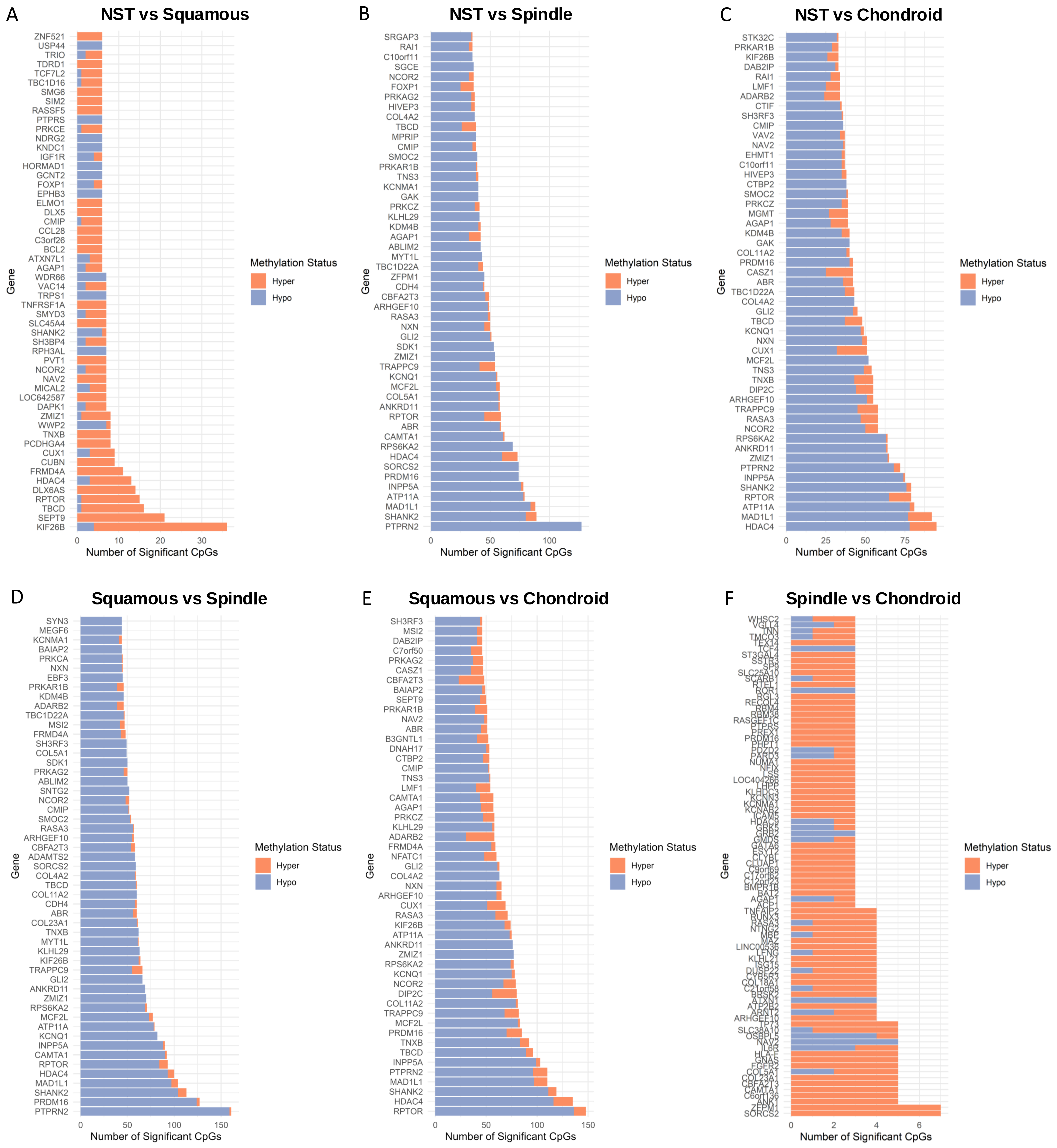

Supplementary Figure 2. Bar plots of the top 50 enriched methylation variable positions (MVPs) for each comparison and their methylation status as hyper or hypo-methylated. The x-axis represents the number of significant MVPs, while the y-axis refers to the enriched genes. 'Hyper' indicates hypermethylation, and 'Hypo' indicates hypomethylation in the "first order" cohort compared to the "second order" cohort. Identified MVPs have $|\Delta\beta| > 0.2$, p-value < 0.01.

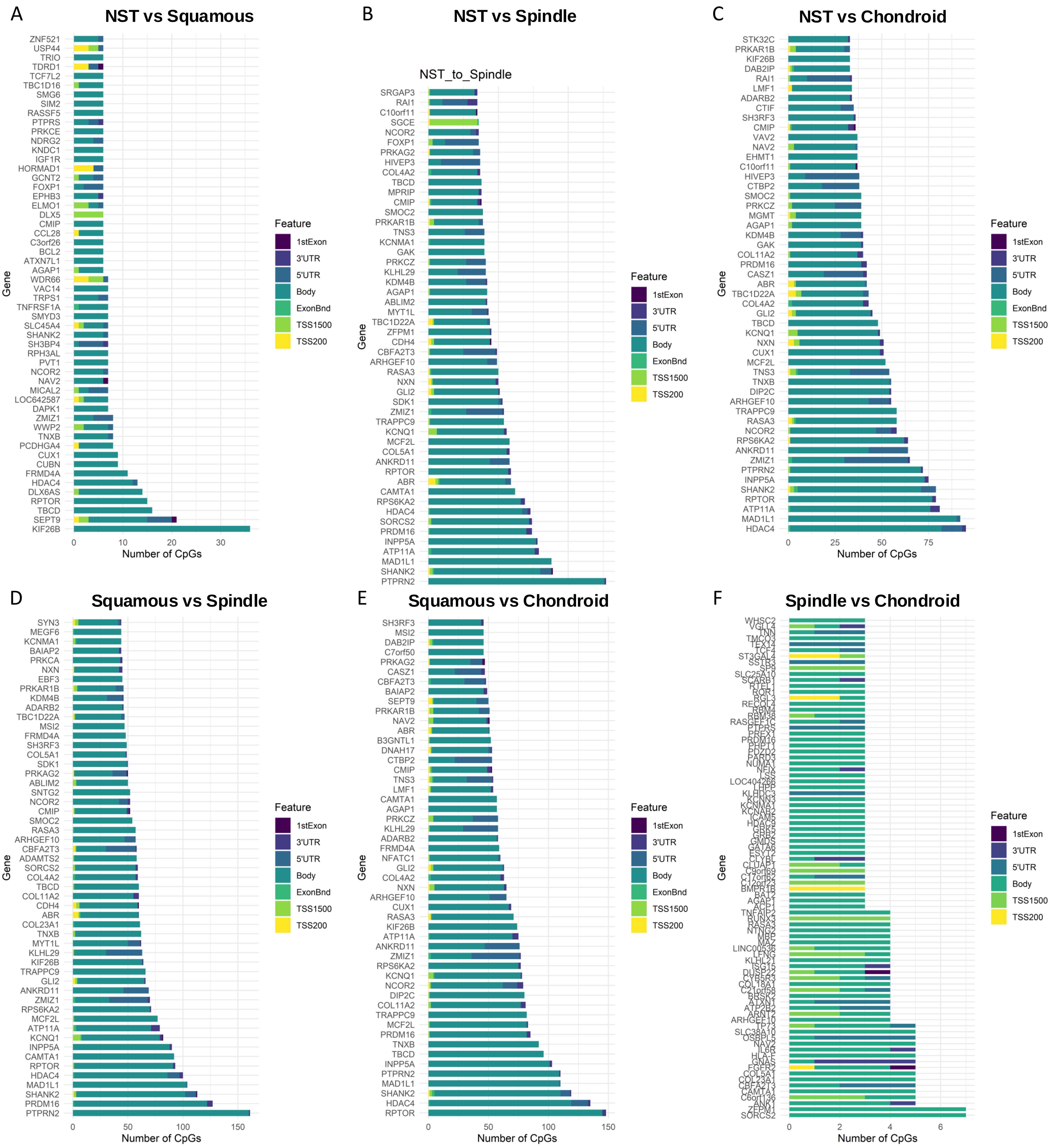

Supplementary Figure 3. Bar plots display the top 50 enriched methylation variable positions (MPVs) for each comparison and their gene localization. The x-axis represents the number of significant MPVs, while the y-axis refers to the enriched genes. Identified MVPs have |Δβ| > 0.2, p-value < 0.01.

A. Number of DMRs between different morphologies

Phenotype	NST	Squamous	Spindle	Chondroid
NST	0	15 DMR	60 DMR	120 DMR
Squamous		0	142 DMR	155 DMR
Spindle			0	21 DMR
Chondroid				0

B. Group distances based on number of DMRs

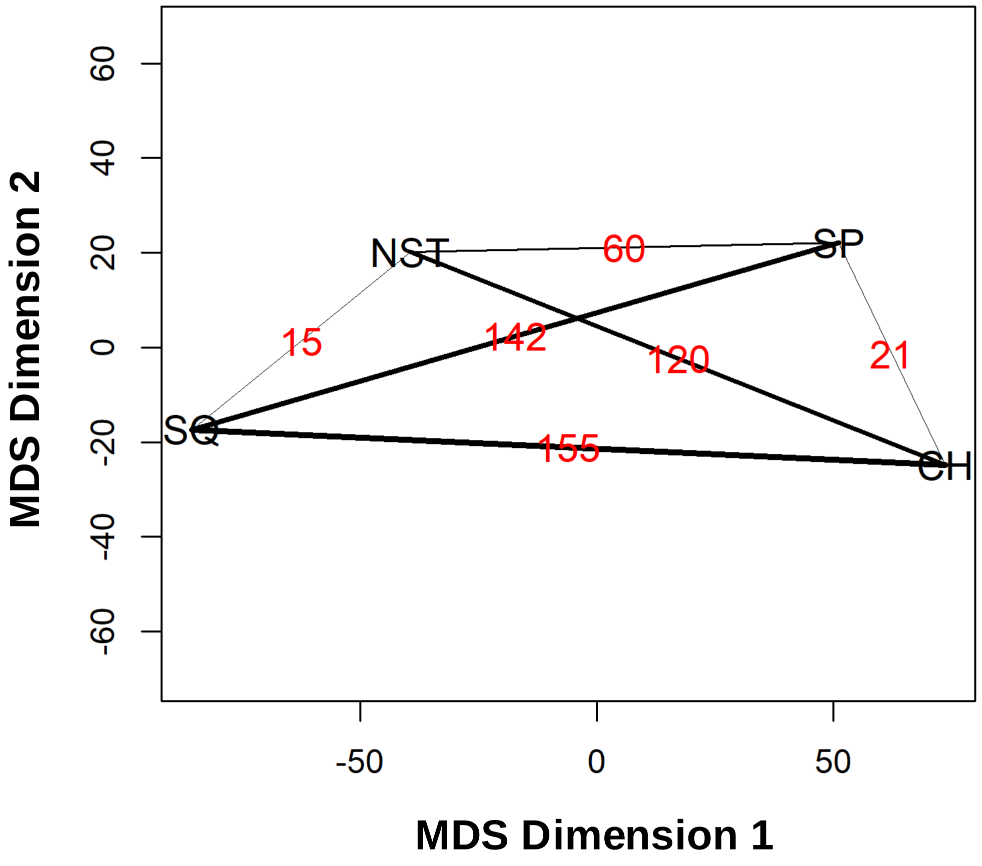

C. The average number of MVPs per gene across different morphologies

Phenotype	NST	Squamous	Spindle	Chondroid
NST	0	1.59 Per gene	3.62 Per gene	3.69 Per genes
Squamous		0	4.34 Per gene	4.55 Per gene
Spindle			0	1.25 Per gene
Chondroid				0

Supplementary Figure 4. Differentially methylated regions (DMRs) and genes between Groups. (A) The number of DMRs between different morphologies. (B) Group distances based on the number of DMRs. The positions of the morphologies were determined using Multidimensional Scaling (MDS), which reduces the complex distance matrix to two dimensions. Red numbers indicate the number of DMRs. The thickness of the connection lines represents the relationship between morphologies, with thinner lines indicating closer relationships. (C) The average number of MVPs per gene across different morphologies.

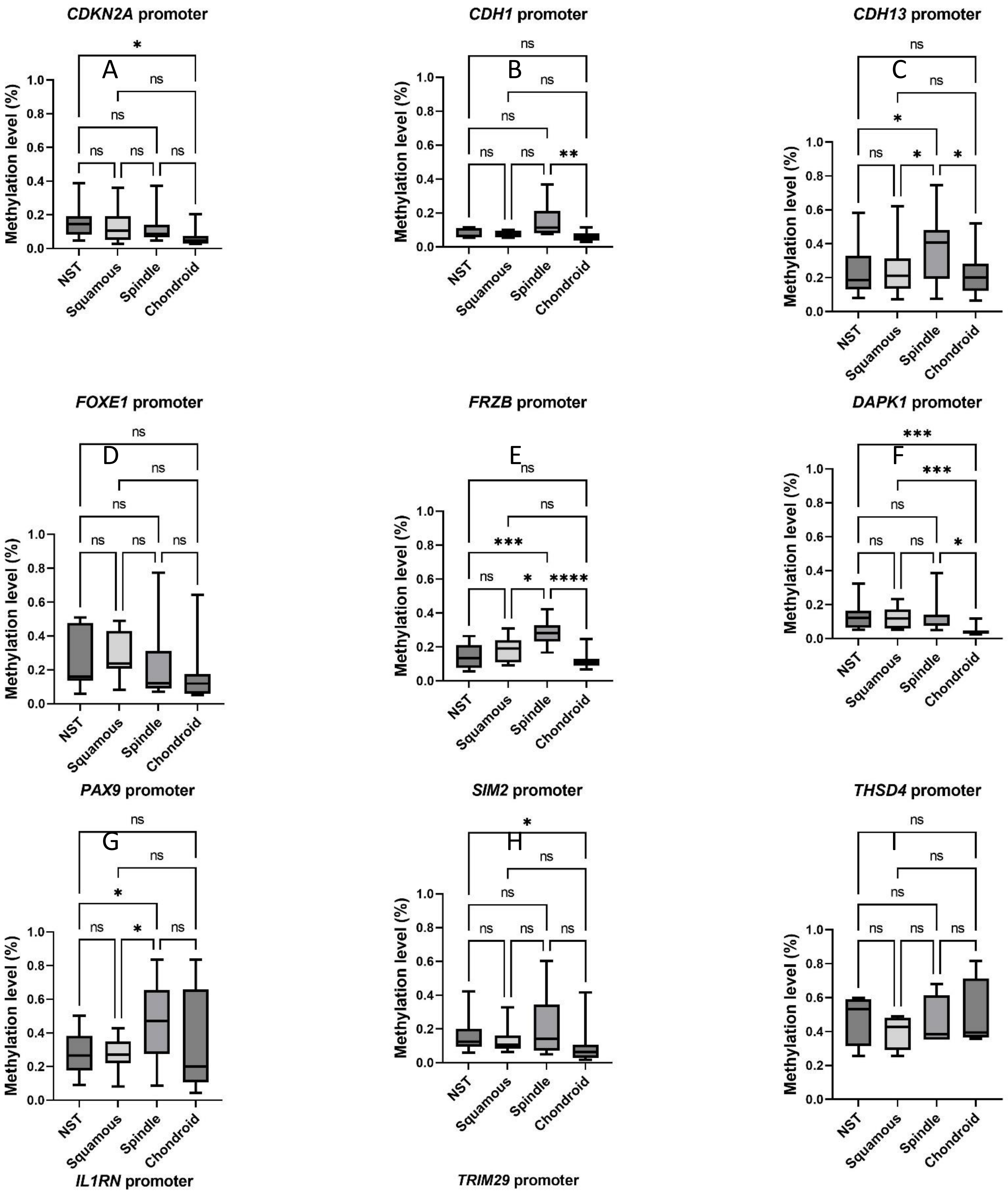

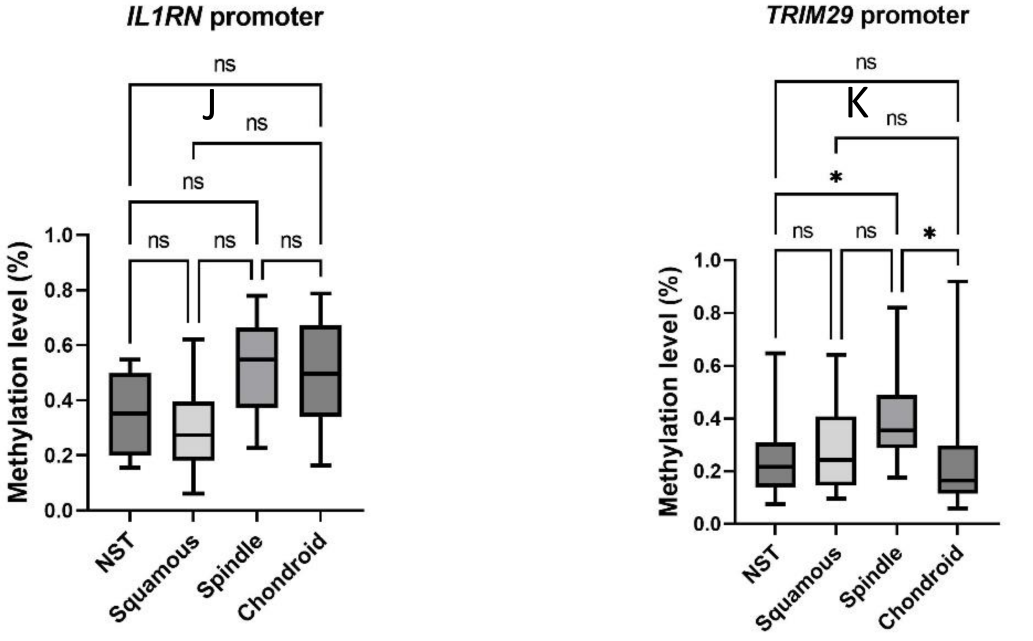

Supplementary Figure 5. Box plot showing the methylation status of key genes identified in cutaneous squamous cell carcinoma (cSCC) and esophageal squamous cell carcinoma (eSCC) across four groups. The x-axis represents different morphologies, and the y-axis represents methylation levels. The red solid line represents the median value, the black dashed line indicates the interquartile range from an early study on TNBC. Kruskal-Wallis tests were used to assess significance between morphological groups. * $P \leq 0.05$; ** $P \leq 0.01$; *** $P \leq 0.001$; **** $P \leq 0.0001$. ns: non-significant.

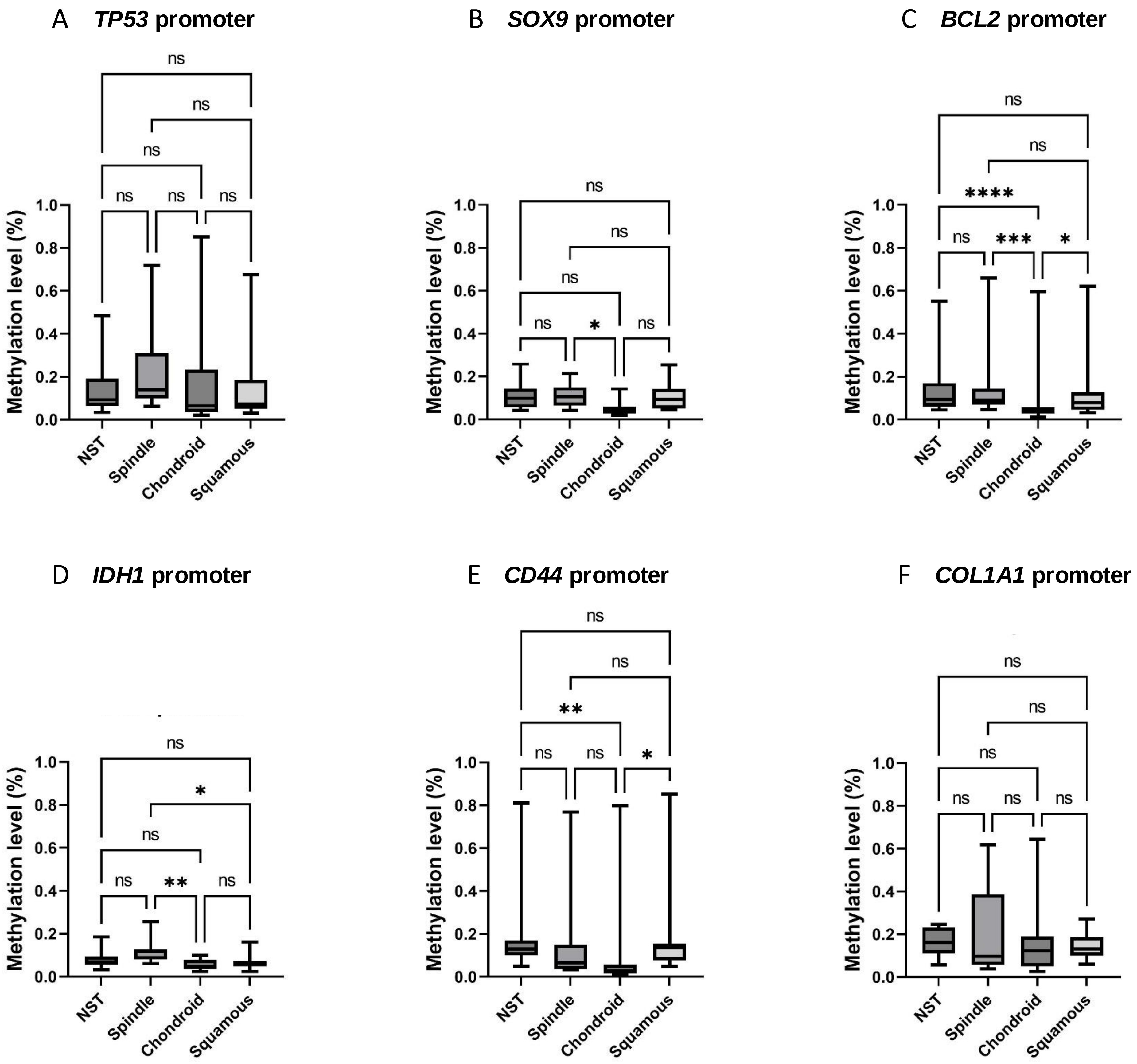

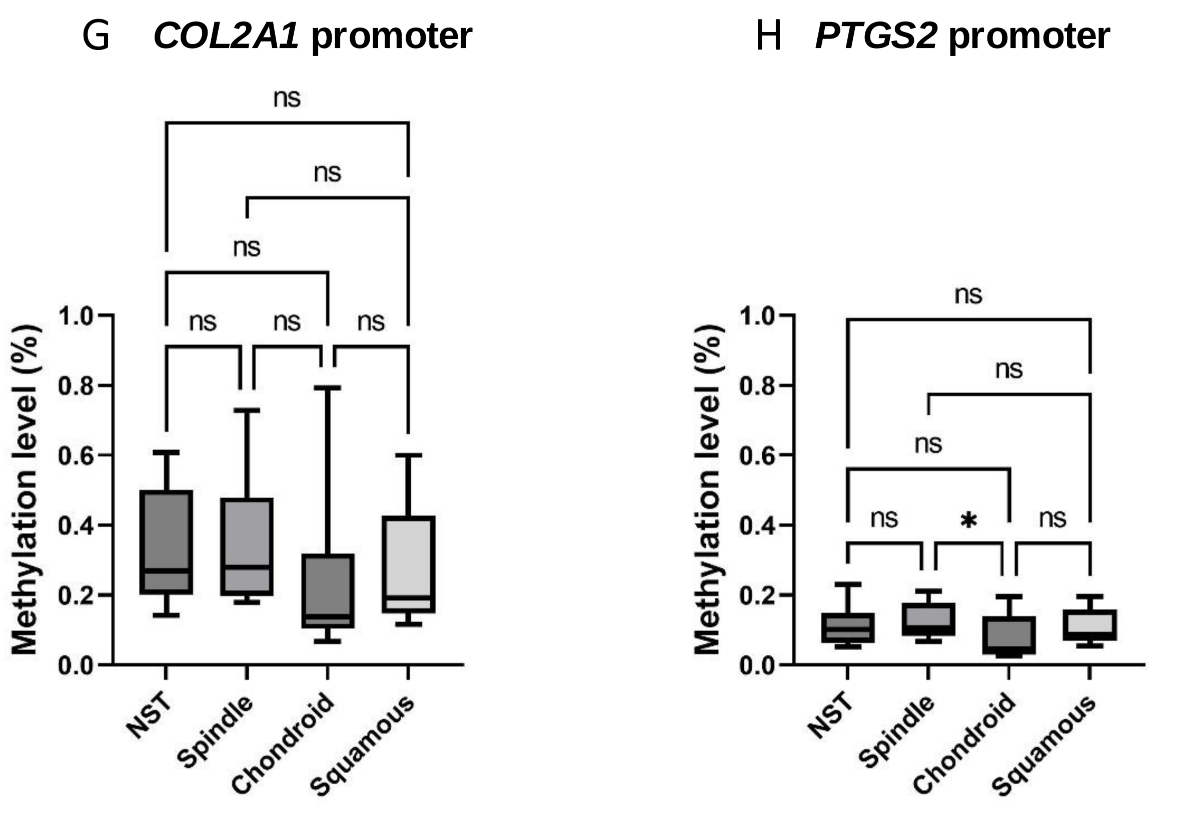

Supplementary Figure 6. Box plot showing the methylation status of key genes identified in chondrosarcoma across four groups. The x-axis represents the different morphologies, the y-axis shows the methylation levels. The line in the middle of the box is plotted at the median. Kruskal-Wallis tests were used to assess significance between morphological groups. * P ≤ 0.05; ** P ≤ 0.01; *** P ≤ 0.001; **** P ≤ 0.0001. ns: non-significant.

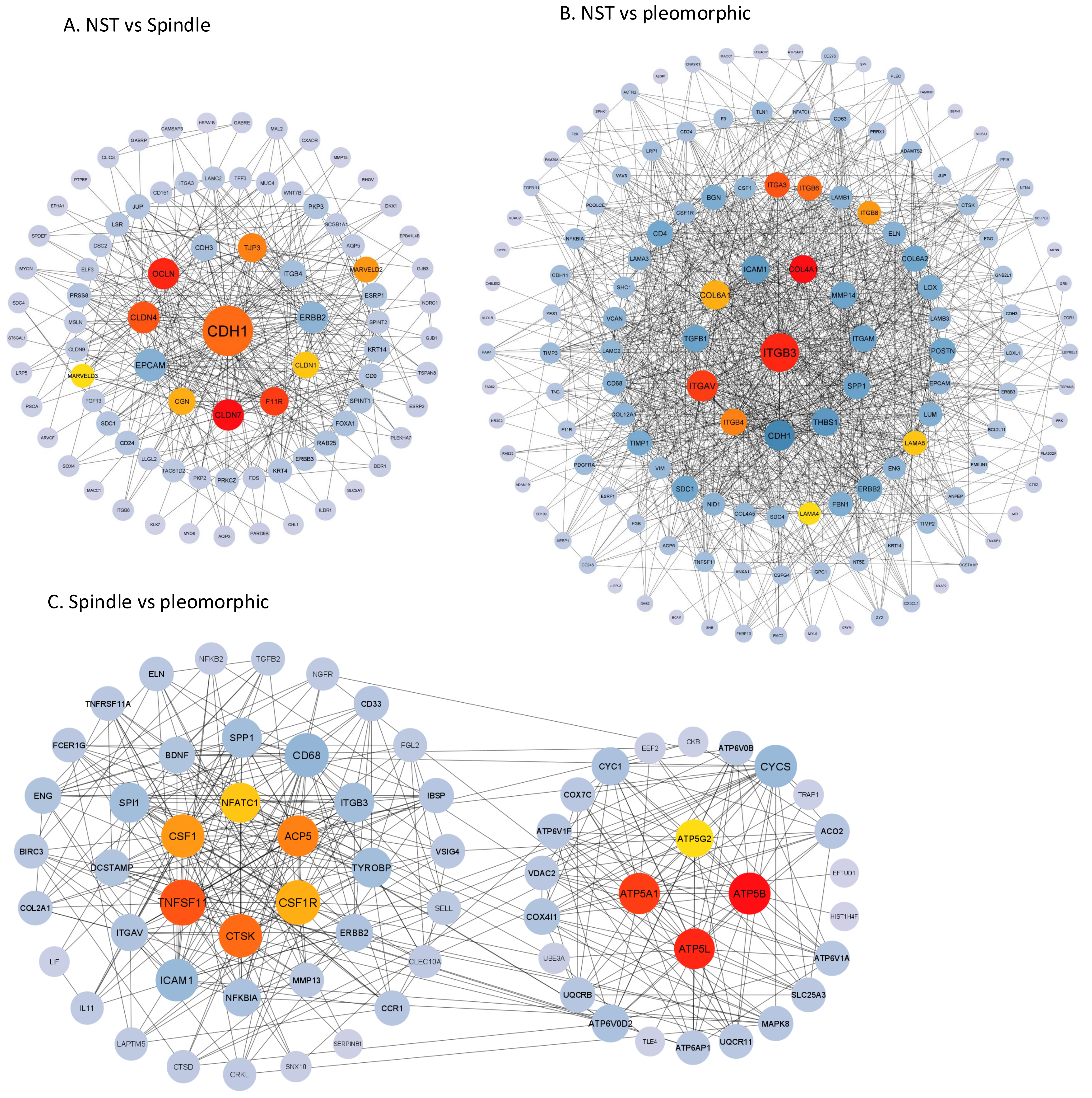

Supplementary Figure 7: Top 10 hub genes and expanded subnetwork across three comparisons in case one. (A) Spindle vs. NST; (B) Spindle vs. Pleomorphic and (C) NST vs. Pleomorphic. The red-to-yellow gradient represents hub genes. With Maximal Clique Centrality (MCC) scores from high (red) to lower (yellow). Genes present in subnetworks are shown in blue.

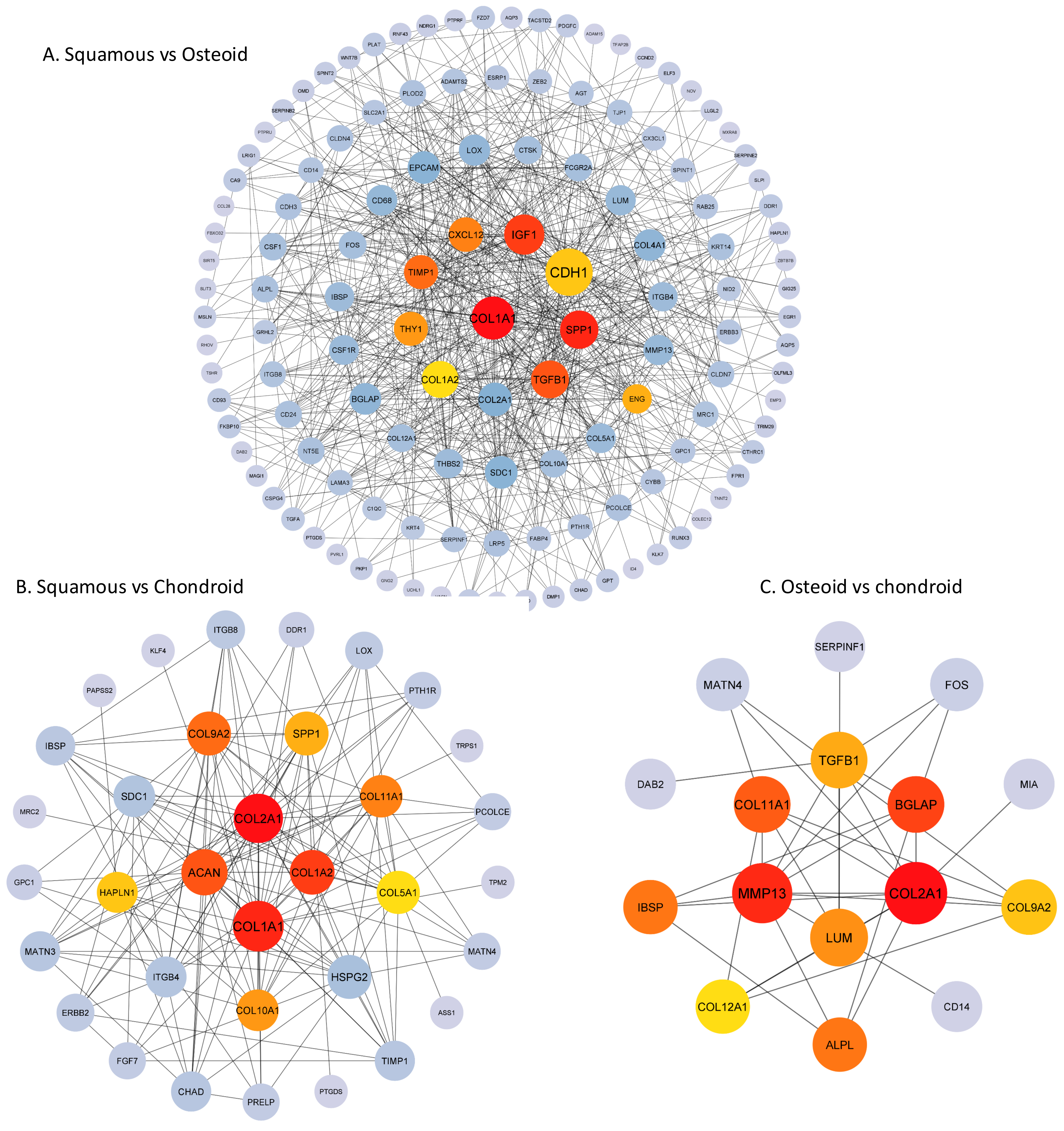

Supplementary Figure 8. Top 10 hub genes and expanded subnetwork across three comparisons in case two. (A) Squamous vs osteoid; (B) Squamous vs. Chondroid (C) Osteoid vs. Chondroid and. The red-to-yellow gradient represents hub genes. With Maximal Clique Centrality (MCC) scores from high (red) to lower (yellow). Genes present in subnetworks are shown in blue.

A. NST vs Chondroid

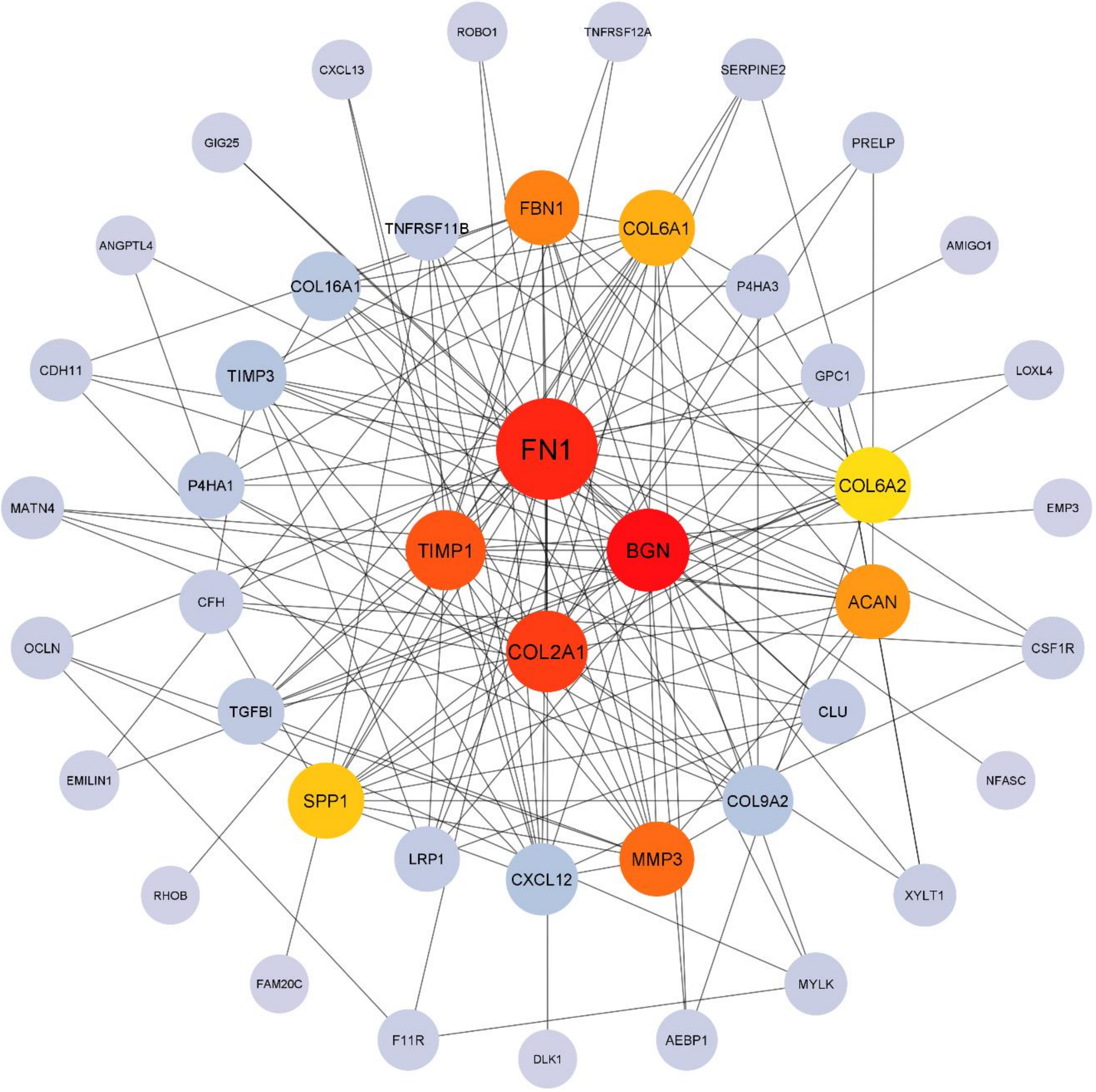

Supplementary Figure 9. Top 10 hub genes and expanded subnetwork for NST vs Chondroid in case three. The red-to-yellow gradient indicates hub genes. With Maximal Clique Centrality (MCC) scores from high (red) to lower (yellow). Genes in subnetworks are displayed in blue.

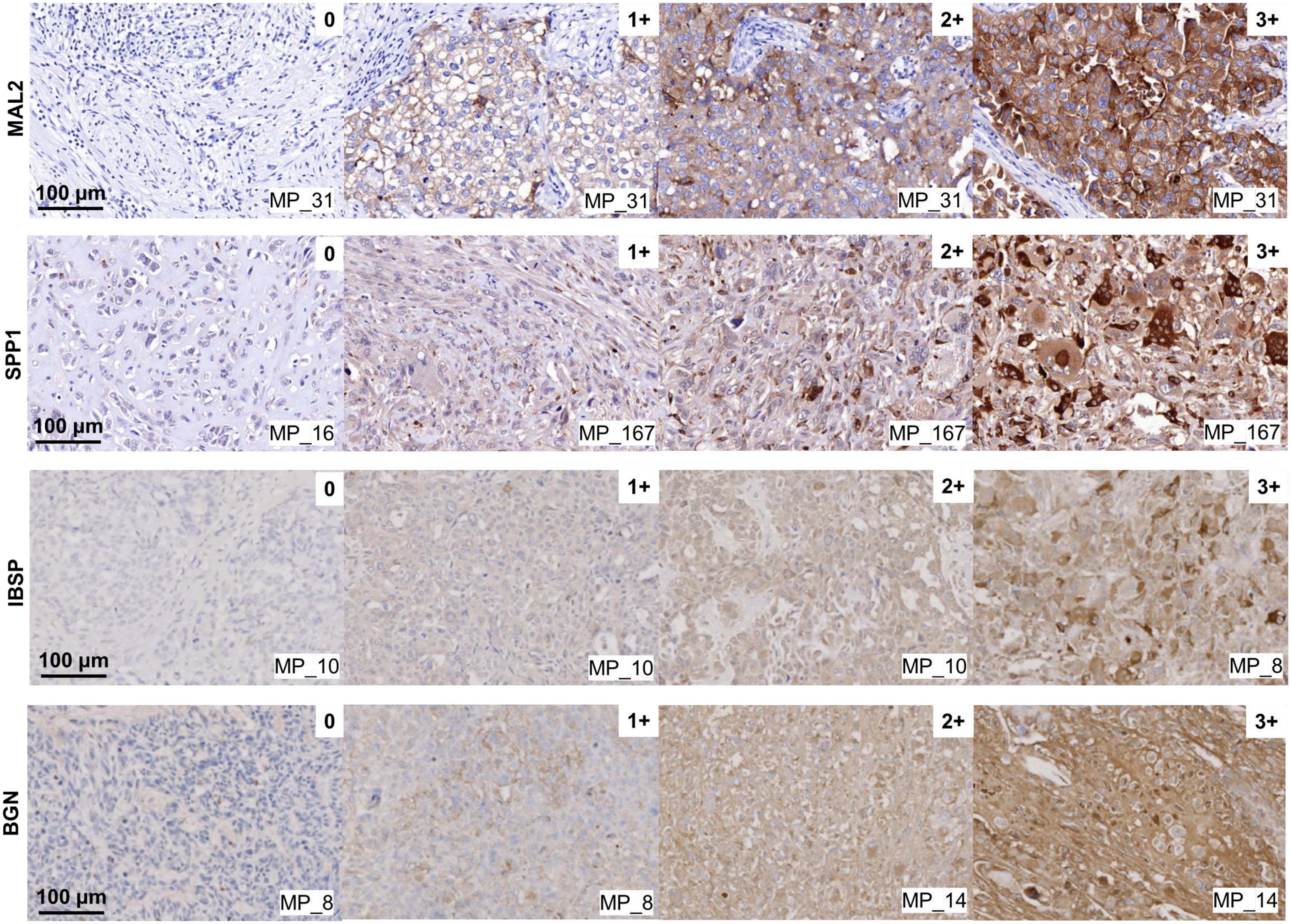

Supplementary Figure 10. Representative Immunohistochemistry (IHC) staining for MAL2, SPP1, IBSP, and BGN, showing staining intensity ranging from 0 (negative) to 3 (strong positive). MAL2 (high in squamous), n=10 samples from 7 cases); SPP1/osteopontin (high in pleomorphic), n=13 from 7 cases; IBSP/bone sialoprotein (high in osteoid) n=7 from 4 cases); and, BGN/biglycan (high in chondroid), n=11 from 8 cases. The image for MAL2 is selected from samples MP_10 (case 2) and MP_31. The image for SPP1 is selected from MP_16 and MP_167 (case 1). The image for IBSP is selected from MP_8 and MP_10 (case 2). The image for BGN is selected from MP_8 and MP_14 (case 3).

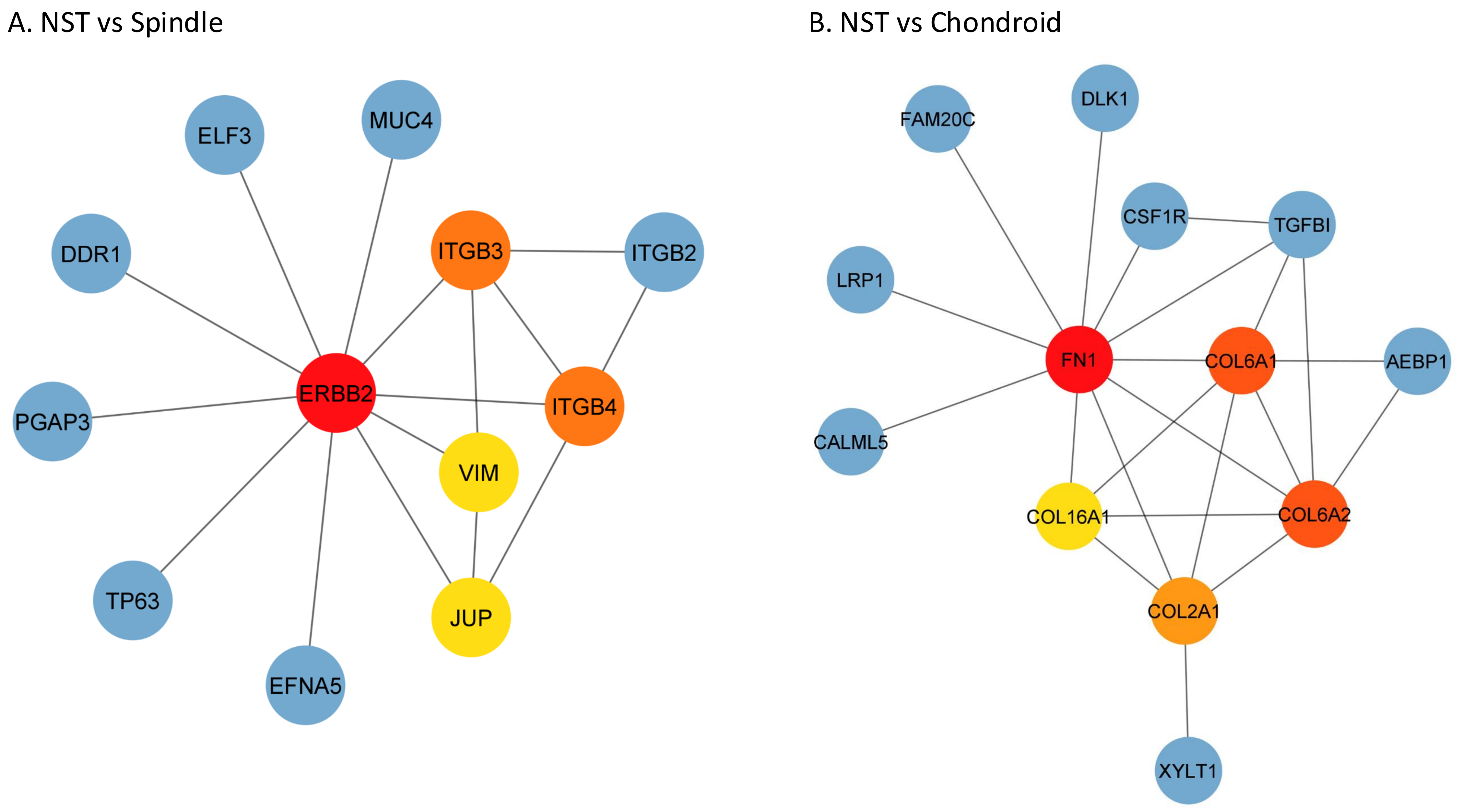

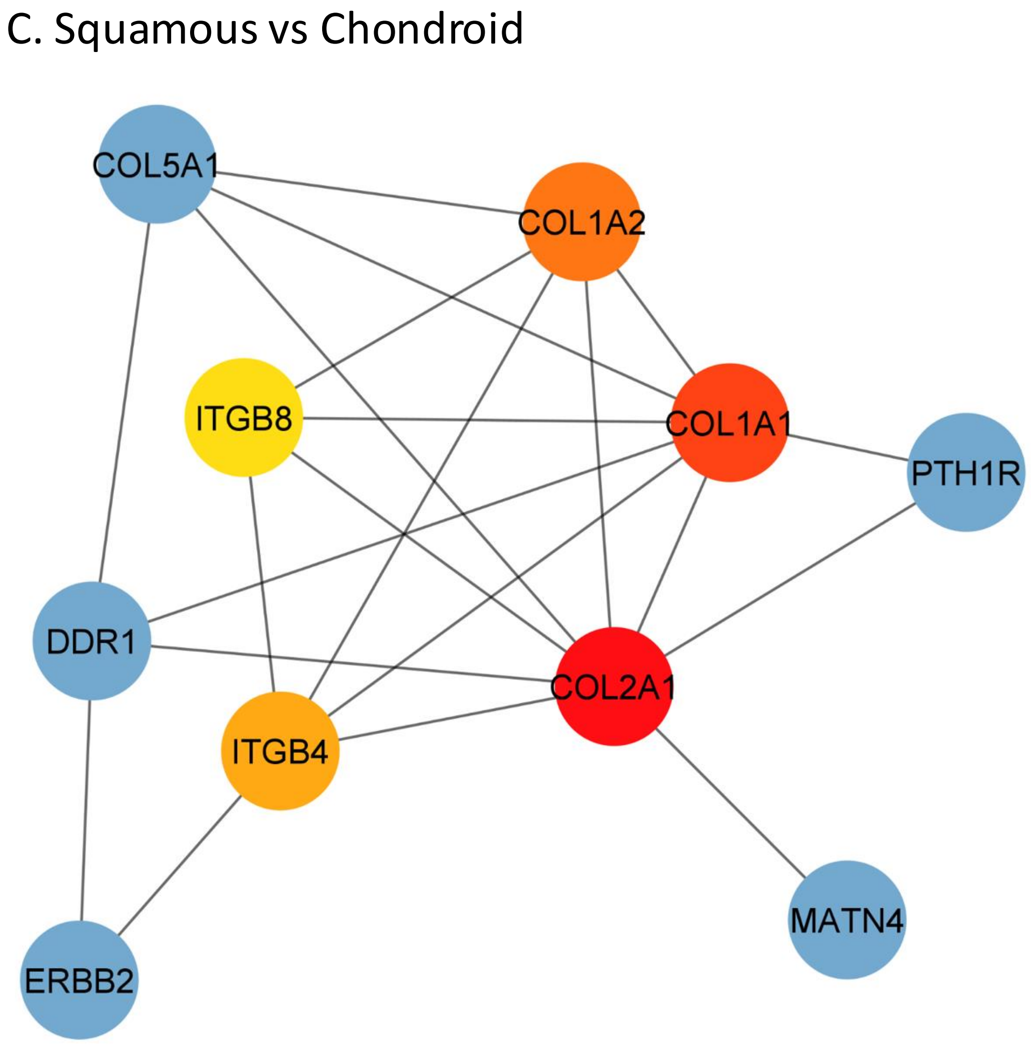

Supplementary Figure 11. Top 5 hub genes for integration analysis. (A) NST vs. Spindle. (B) NST vs. Chondroid. (C) Squamous vs. Chondroid. The red-to-yellow gradient indicates hub genes, genes in subnetworks are displayed in blue.

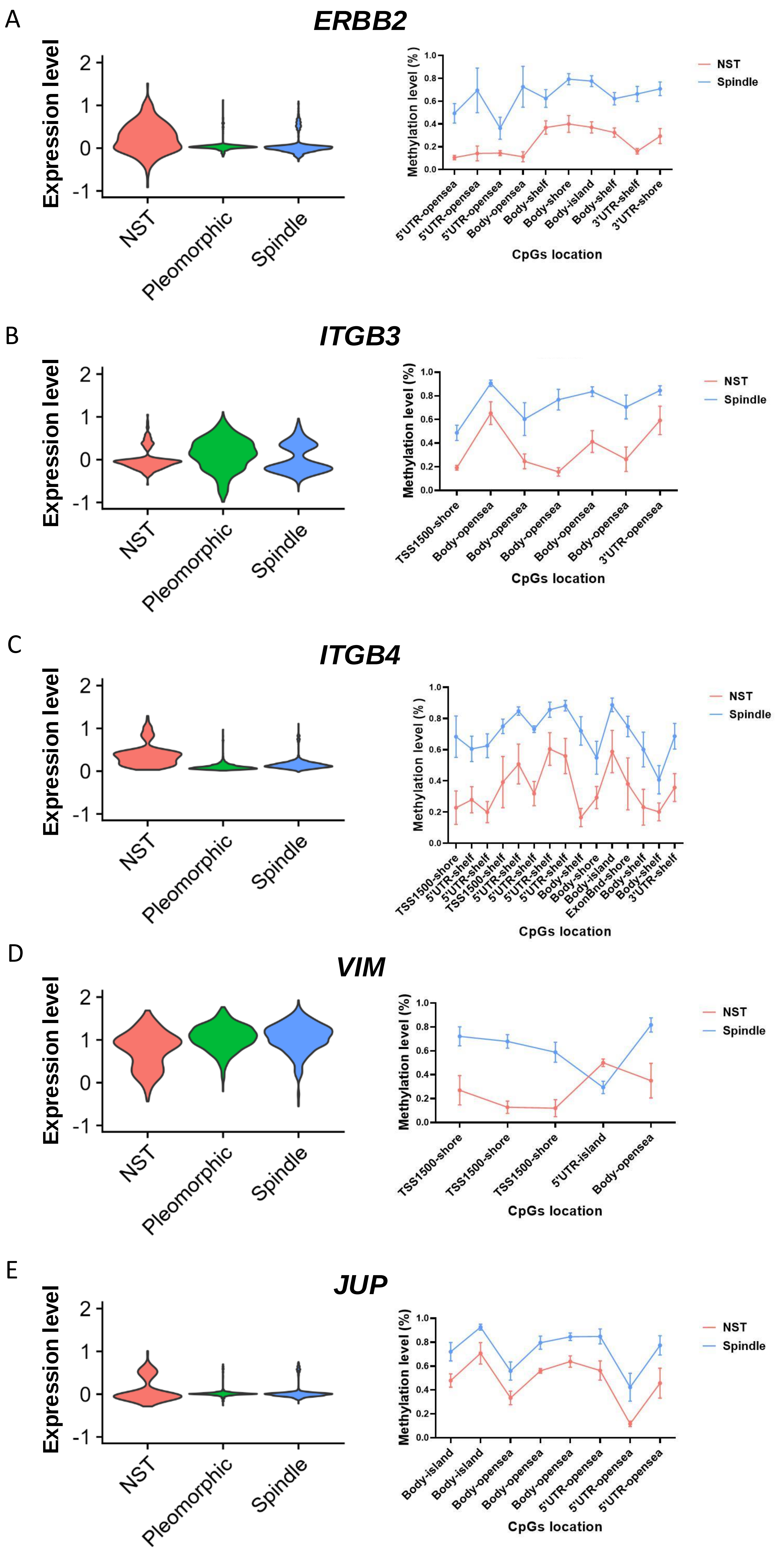

Supplementary Figure 12. Gene expression and methylation patterns for key genes identified in the integration analysis for NST vs. Spindle comparison. The left-side plots show transcriptomic expression levels in Case one (MP_167) for the top five hub genes: (A) ERBB2, (B) ITGB3, (C) ITGB4, (D) VIM, and (E) JUP. The right-side plots show methylation patterns for MVPs ($|\Delta\beta| > 0.2$, p-value < 0.01) identified in the NST vs. Spindle comparison.

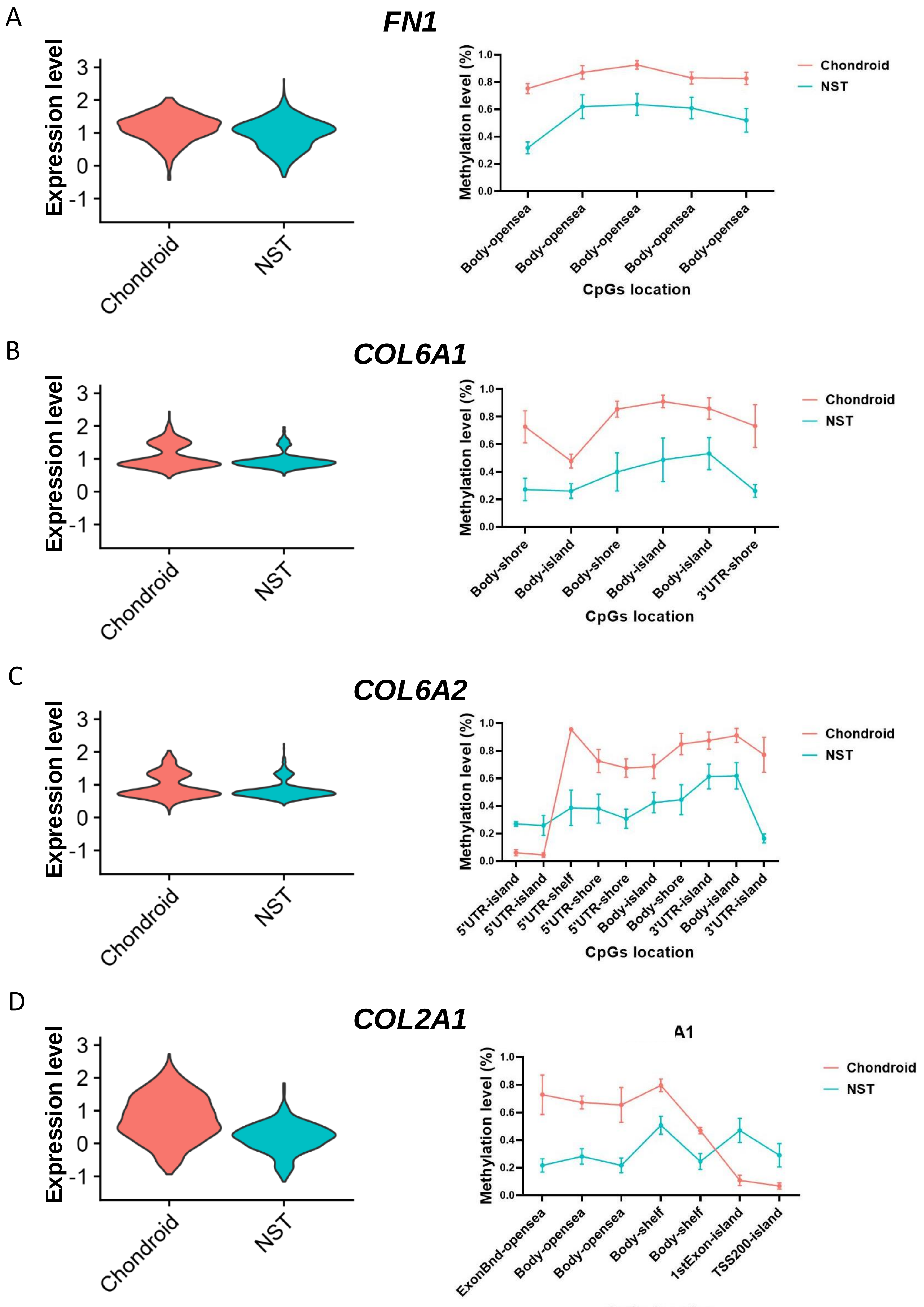

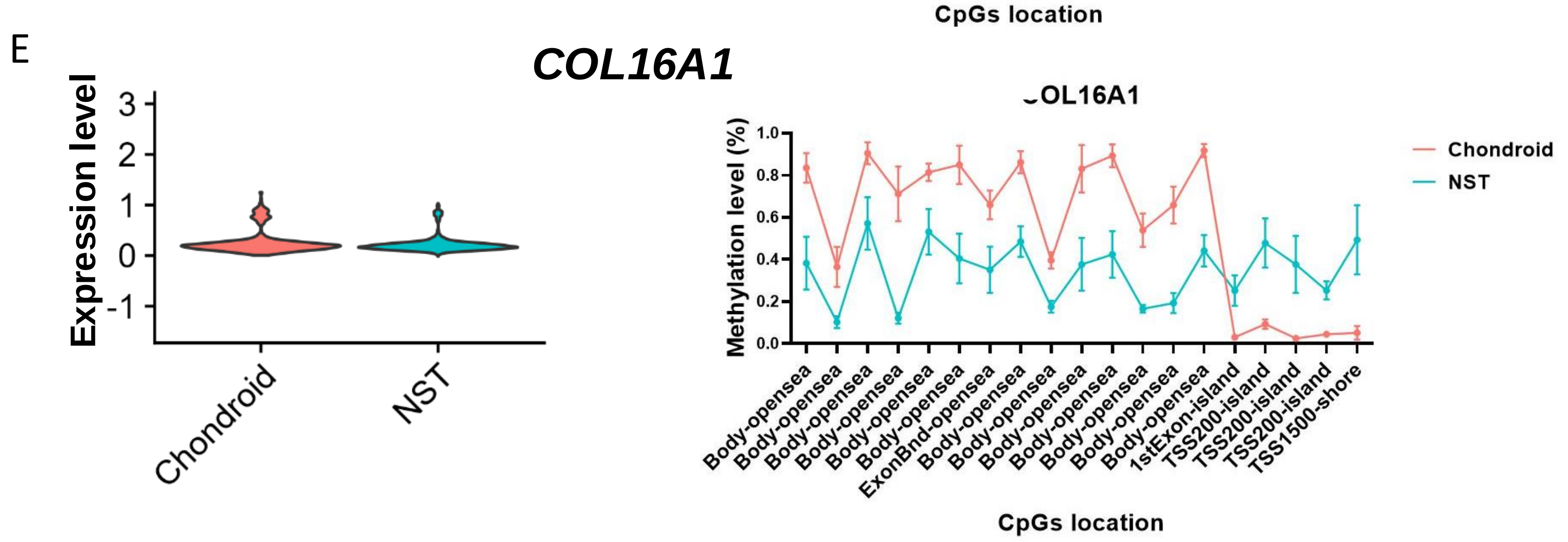

Supplementary Figure 13. Gene expression and methylation patterns for key genes identified in the integration analysis for NST vs. Chondroid comparison. The left-side plots show transcriptomic expression levels in Case three (MP_10) for the top five hub genes: (A) FN1, (B) COL6A1, (C) COL6A2, (D) COL2A1, and (E) COL16A1. The right-side plots show methylation patterns for MVPs ($|\Delta\beta|$ >0.2, p-value < 0.01) identified in the NST vs. Chondroid comparison.

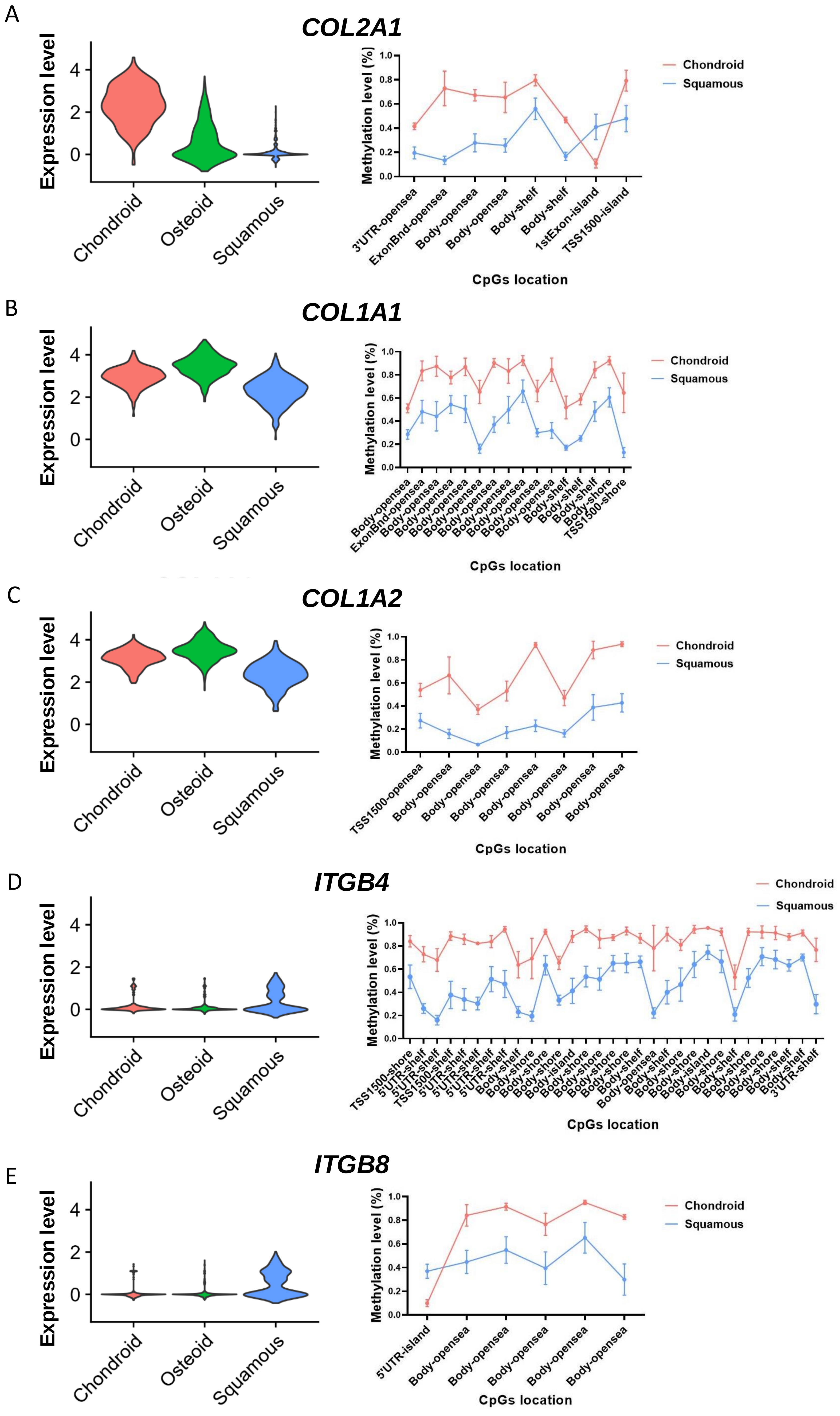

Supplementary Figure 14. Gene expression and methylation patterns for key genes identified in the integration analysis for Squamous vs. Chondroid comparison. The left-side plots show transcriptomic expression levels in Case two (MP_10) for the top five hub genes: (A) COL2A1, (B) COL1A1, (C) COL1A2, (D) ITGB4, and (E) ITGB8. The right-side plots show methylation patterns for MVPs ($|\Delta\beta| > 0.2$, p-value < 0.01) identified in the Squamous vs. Chondroid comparison.

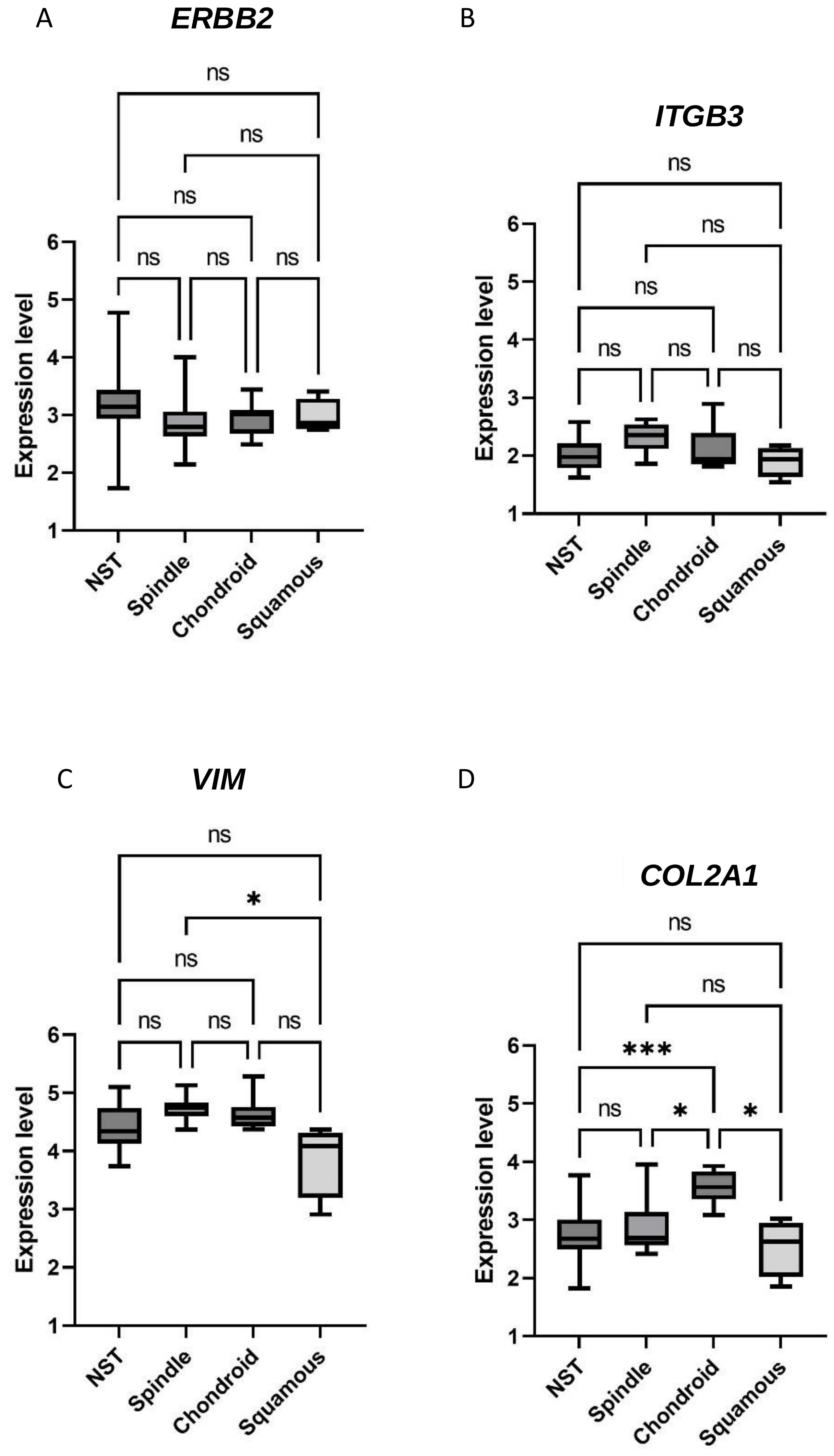

Supplementary Figure 15. Expression levels across various metaplastic components. The x-axis represents different morphologies, and the y-axis reflects expression level. The line in the middle of the box is plotted at the median. Data accessed from (GSE212245). Kruskal-Wallis tests were used to assess significance between morphological groups. * P ≤ 0.05; ** P ≤ 0.01; *** P ≤ 0.001. ns: non-significant.

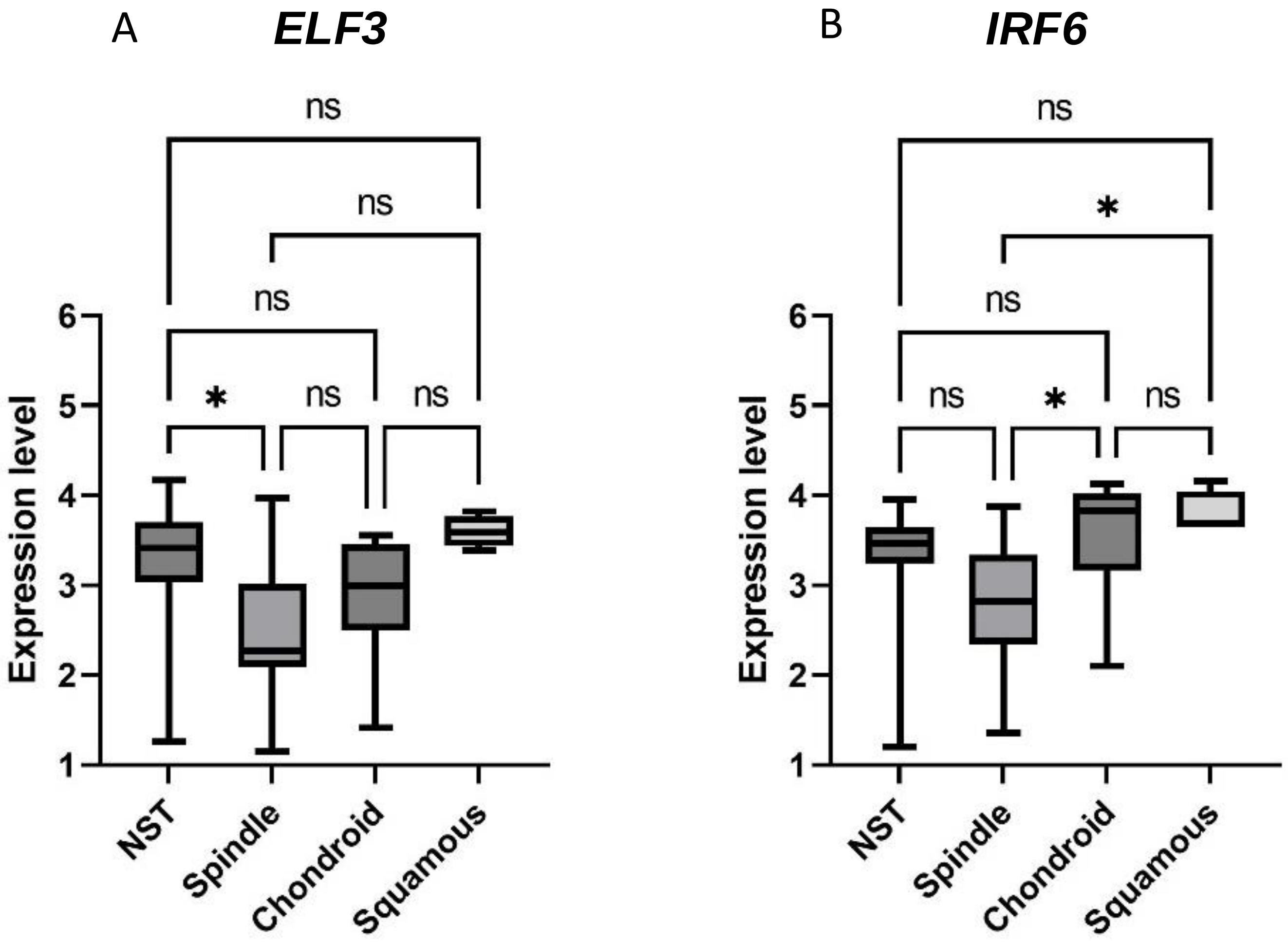

Supplementary Figure 16. Expression levels of ELF3 and IRF6 across various metaplastic components. The x-axis represents different morphologies, and the y-axis reflects expression level. The line in the middle of the box is plotted at the median. Data accessed from (GSE212245). The Kruskal-Wallis tests were used to assess significance between morphological groups. An asterisk (*) indicates P ≤ 0.05. ns: non-significant.

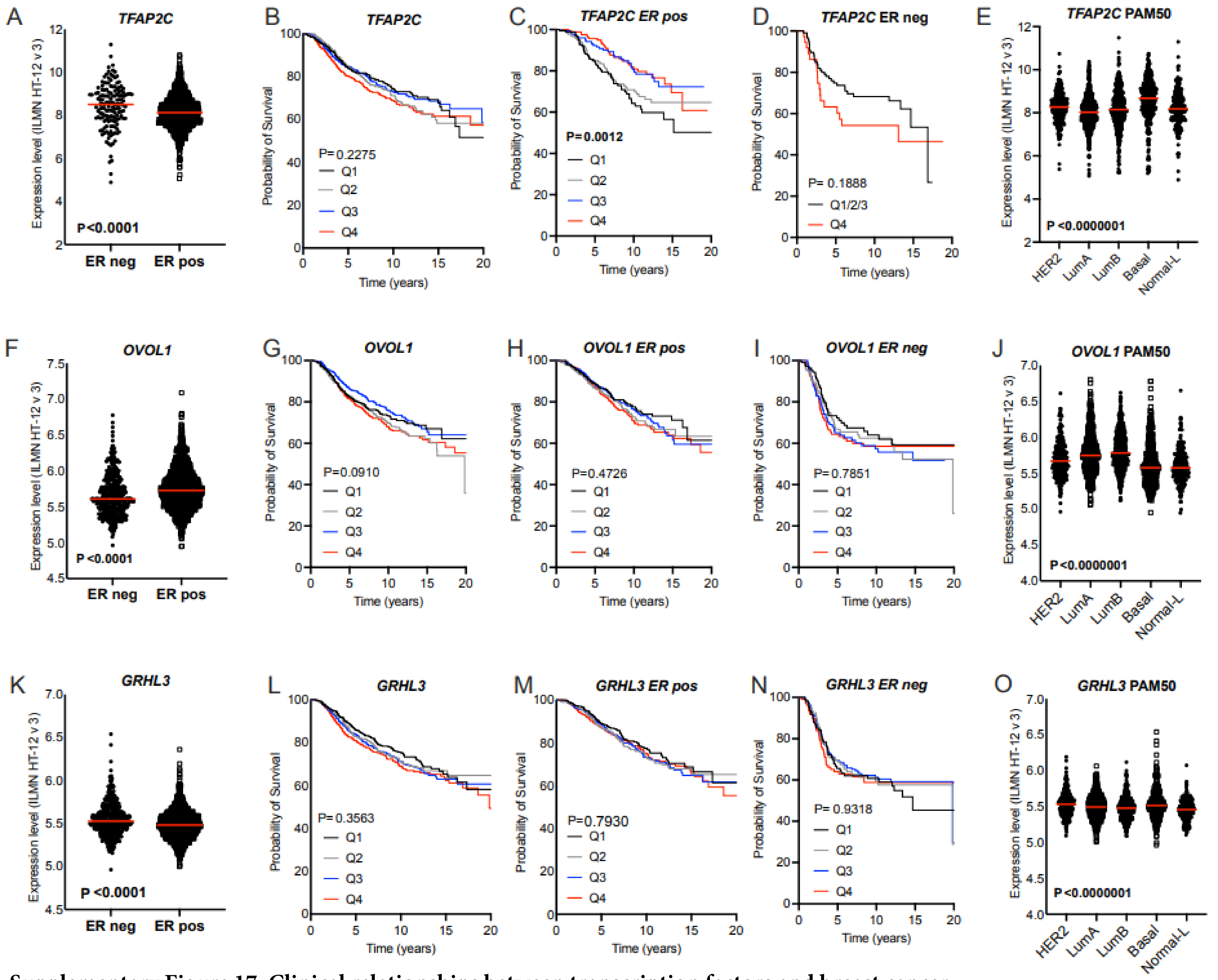

Supplementary Figure 17. Clinical relationships between transcription factors and breast cancer. (A) TFAP2C expression level comparison between ER positive and ER negative breast cancers (Mann Whitney U: P<0.0001). Kaplan Meier curve survival analysis (Log-rank test) of TFAP2C expression split by quartiles (where Q1 is lowest and Q4 is highest expression) in the whole cohort (B), ER positive (C) and ER negative (D) breast cancers. (E) TFAP2C expression level comparison across PAM50 subtypes (ANOVA, Welch's; P<0.00000001). (F) OVOL1 expression level comparison between ER positive and ER negative breast cancers (Mann Whitney U: P<0.0001). Kaplan Meier curve survival analysis (Log-rank test) of OVOL1 expression split by quartiles (where Q1 is lowest and Q4 is highest expression) in the whole cohort (G), ER positive (H) and ER negative (I) breast cancers. (J) OVOL1 expression level comparison across PAM50 subtypes (ANOVA, Welch's; P<0.00000001). (K) GRHL3 expression level comparison between ER positive and ER negative breast cancers (Mann Whitney U: P<0.0001). Kaplan Meier curve survival analysis (Log-rank test) of GRHL3 expression split by quartiles (where Q1 is lowest and Q4 is highest expression) in the whole cohort (L), ER positive (M) and ER negative (N) breast cancers. (O) GRHL3 expression level comparison across PAM50 subtypes (ANOVA, Welch's; P<0.00000001).

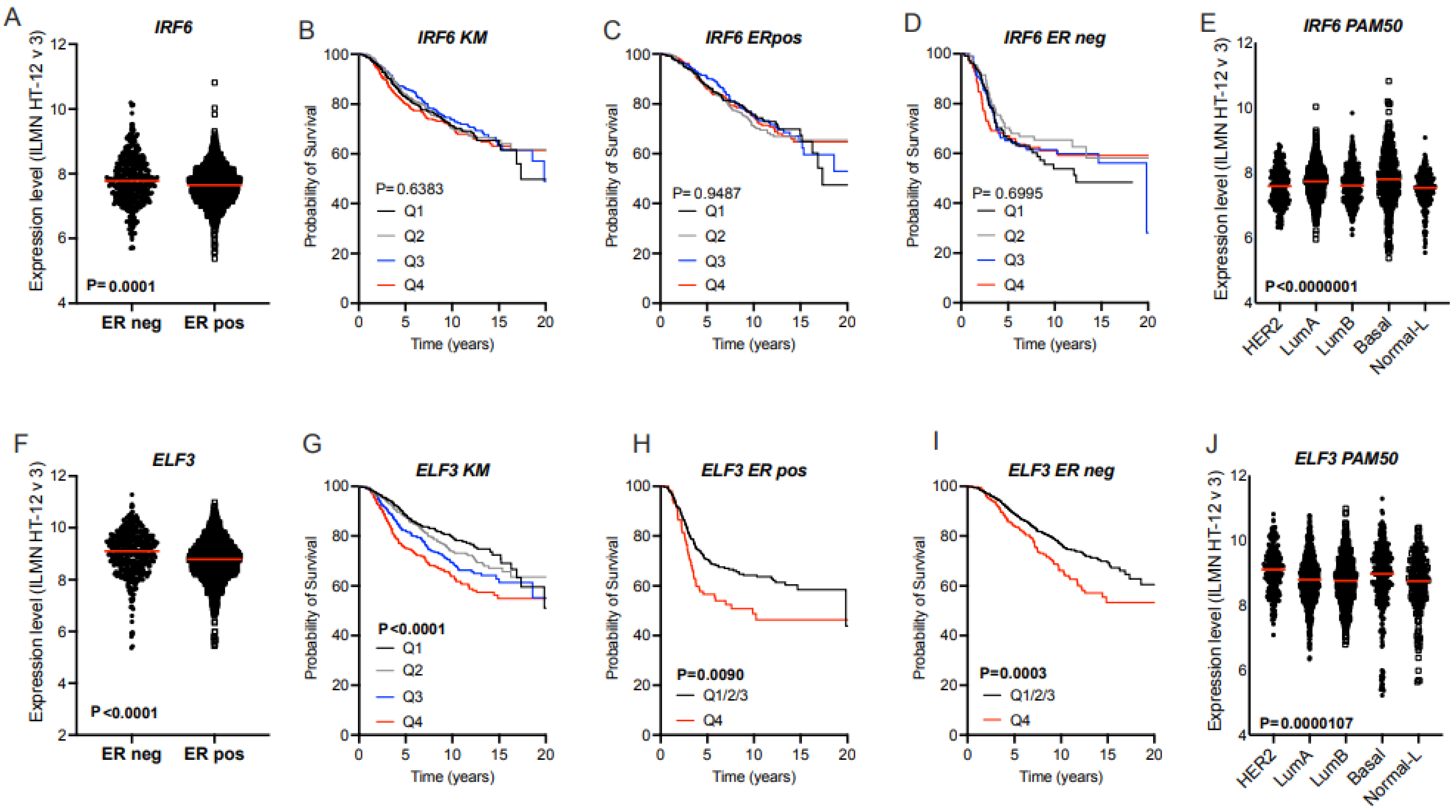

Supplementary Figure 18. Clinical relationships between transcription factors and breast cancer. (A) IRF6 expression level comparison between ER positive and ER negative breast cancers (Mann Whitney U: P<0.0001). Kaplan Meier curve survival analysis (Log-rank test) of IRF6 expression split by quartiles (where Q1 is lowest and Q4 is highest expression) in the whole cohort (B), ER positive (C) and ER negative (D) breast cancers. (E) IRF6 expression level comparison across PAM50 subtypes (ANOVA, Welch's; P<0.00000001). (F) ELF3 expression level comparison between ER positive and ER negative breast cancers (Mann Whitney U: P<0.0001). Kaplan Meier curve survival analysis (Log-rank test) of ELF3 expression split by quartiles (where Q1 is lowest and Q4 is highest expression) in the whole cohort (G), ER positive (H) and ER negative (I) breast cancers. (J) ELF3 expression level comparison across PAM50 subtypes (ANOVA, Welch's; P<0.00000001).

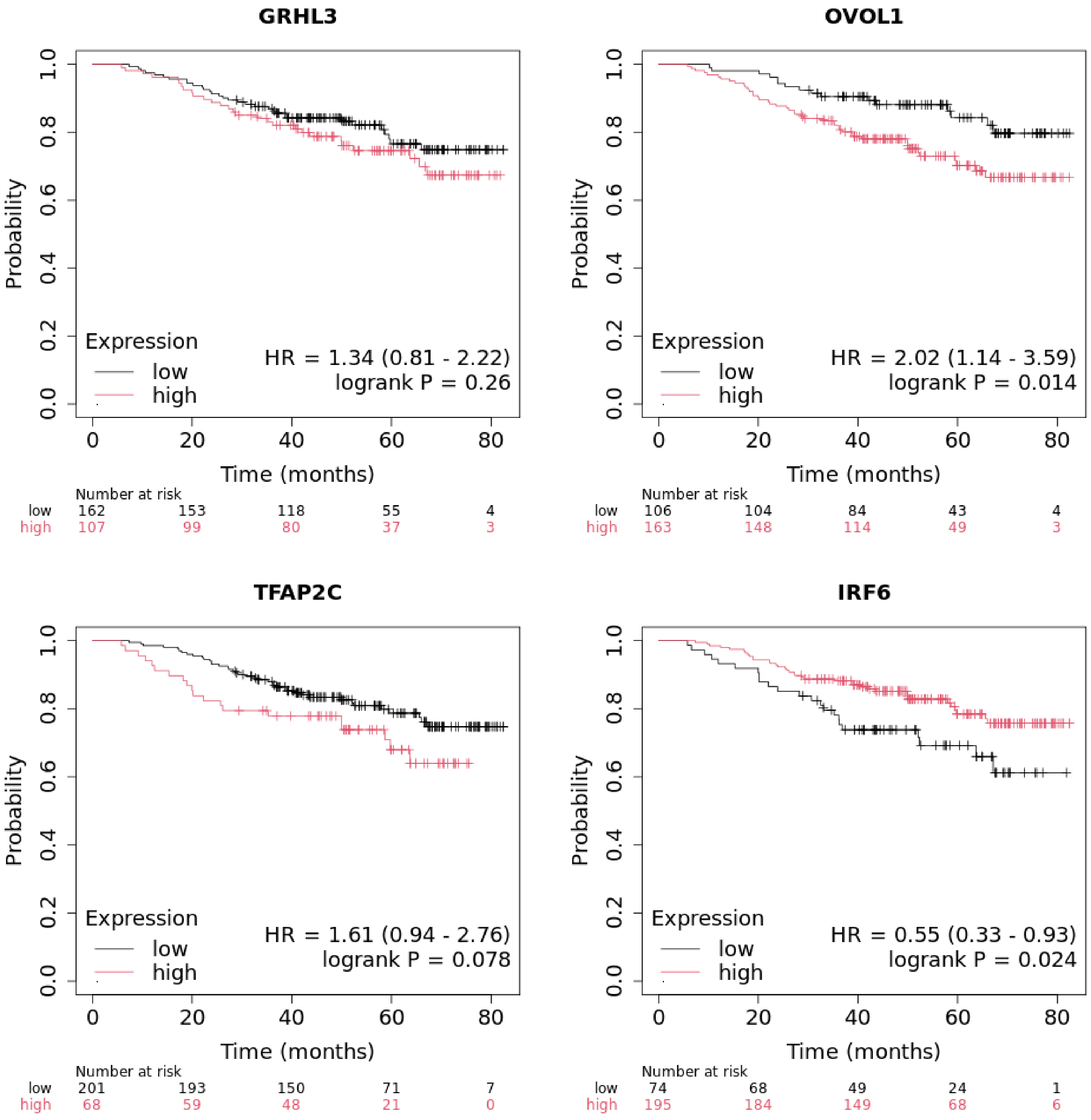

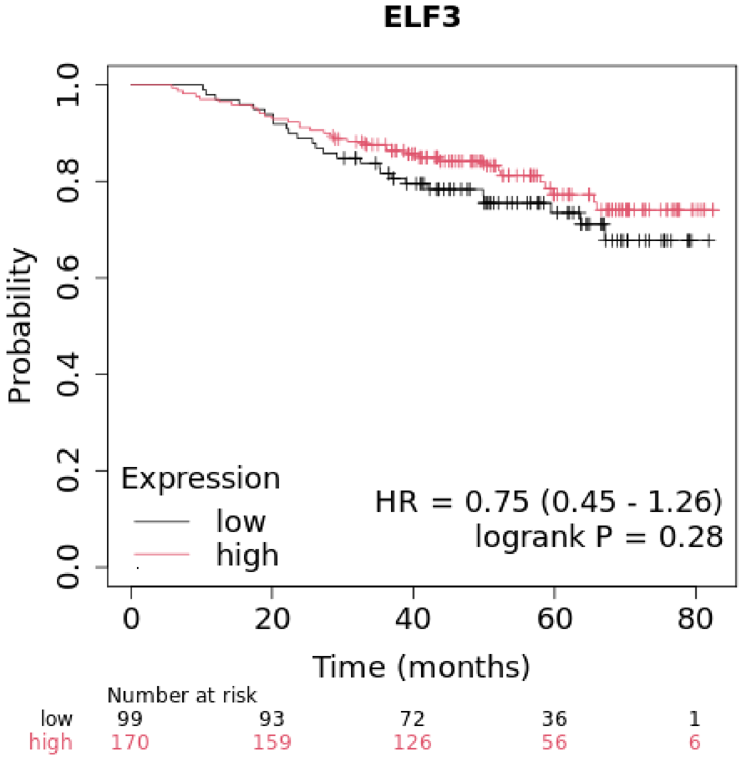

Supplementary Figure 19. Kaplan-Meier (KM) curve survival analysis using the KMPlotter dataset. Cases were selected that were basal and grade 3 – the most metaplastic-like features. The cohort was split on the auto cut-off setting to enhance survival relationships for (A) GRHL3, (B) OVOL1, (C) TFAP2C, (D) IRF6 and, (E) ELF3. Log rank P value as noted.
